# Unusual Excimer/Dimer Behavior of a Highly Soluble C,N Platinum(II) Complex with a Spiro-Fluorene Motif

**DOI:** 10.1021/acs.inorgchem.3c02667

**Published:** 2023-10-31

**Authors:** Piotr Pander, Andrey V. Zaytsev, Larissa Gomes Franca, Fernando B. Dias, Valery N. Kozhevnikov

**Affiliations:** † Faculty of Chemistry, Silesian University of Technology, Strzody 9, 44-100 Gliwice, Poland; ‡ Centre for Organic and Nanohybrid Electronics, Silesian University of Technology, Konarskiego 22B, 44-100 Gliwice, Poland; § Department of Applied Sciences, Faculty of Health and Life Sciences, Northumbria University, Newcastle Upon Tyne NE1 8ST, Tyne and Wear, U.K.; ∥ Department of Physics, Durham University, South Road, Durham DH1 3LE, U.K.; ⊥ Department of Materials Science and Metallurgy, University of Cambridge, Cambridge CB3 0FS, U.K.

## Abstract

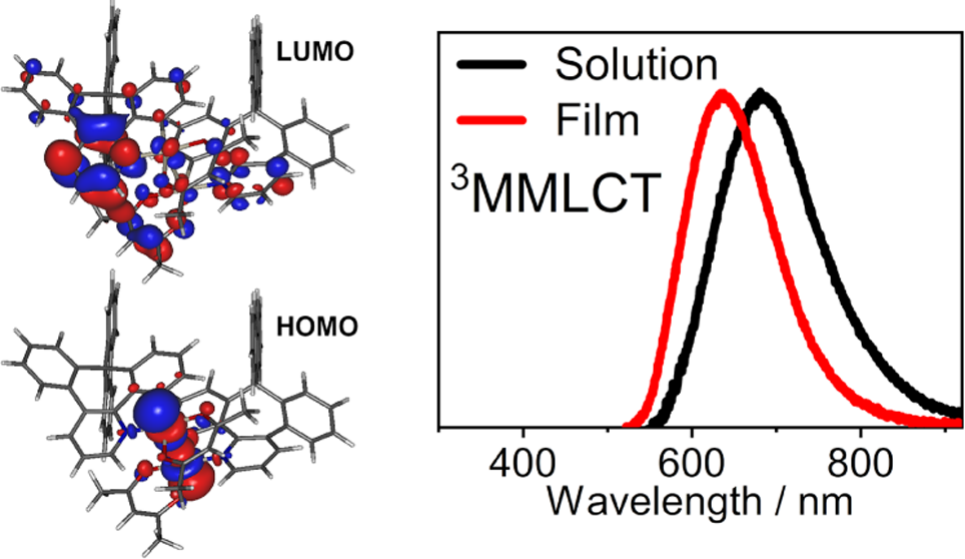

In this work, we introduce a spiro-fluorene unit into a phenylpyridine (CN)-type ligand as a simple way to deplanarize the structure and increase the solubility of the final platinum(II)···complex. Using a spiro-fluorene unit, orthogonal to the main coordination plane of the complex, reduces intermolecular interactions, leading to increased solubility but without significantly affecting the ability of the complex to form Pt···Pt dimers and excimers. This approach is highly important in the design of platinum(II) complexes, which often suffer from low solubility due to their mainly planar structure, and offers an alternative to the use of bulky alkyl groups. The nonplanar structure is also beneficial for vacuum-deposition techniques as it lowers the sublimation temperature. Importantly, there are no sp^3^ hybridized carbon atoms in the cyclometalating ligand that contain hydrogens, the undesired feature that is associated with the low stability of the materials in OLEDs. The complex displays high solubility in toluene, ∼10 mg mL^–1^, at room temperature, which allows producing solution-processed OLEDs in a wide range of doping concentrations, 5–100%, and EQE up to 5.9%, with a maximum luminance of 7400 cd m^–2^. Concurrently, we have also produced vacuum-deposited OLEDs, which display luminance up to 32 500 cd m^–2^ and a maximum EQE of 11.8%.

## Abstract 2

A novel, highly soluble platinum(II) complex displays unusual behavior of the excimer/dimer ^3^MMLCT emission in solid films.

## Introduction

Low solubility or high susceptibility to aggregation is a common feature of planar luminophores, such as platinum(II) complexes^1−3^ or multiple-resonance (MR) thermally activated delayed fluorescence (TADF) emitters^4^ for example. This behavior originates from relatively strong interplanar π···π interactions, which in planar structures are completely undisturbed. If high solubility or low aggregation is desired, the typical way to tackle the problem is by decorating the structure of luminophores with linear or branched alkyls,^5,6^ cycloalkene units,^7,8^ or through the use of aromatic rings orthogonal to the main luminophore plane, such as mesitylene^9^ or diisopropylphenyl.^10^ A possible disadvantage of this approach is the appearance of additional rotavibrational motions associated with these groups, leading to a potential luminescence quenching due to the enhanced nonradiative decay. The use of a spiro-linked fluorene unit orthogonal to the main coordination plane of the platinum complex poses an interesting approach in which the rigid aromatic groups are fixed in position. This design features a relatively rigid spiro linkage through a quaternary sp^3^ carbon atom that not only reduces the nonradiative decay but also eliminates conformational disorder, leading to a more favorable behavior of the luminophore in solid films and OLEDs.^11,12^

The low solubility of platinum(II) complexes poses a significant limitation in synthesis and characterization, as well as on their use as luminescent dopants in organic light-emitting diodes (OLEDs). This is because strong intermolecular interactions, originating from π···π and Pt···Pt contacts, are likely to be affecting their electronic properties.^13−15^ This issue may be of even greater importance for diplatinum(II) complexes, where the planar conjugated structure is extended. These complexes are of particular interest due to their strong near-infrared (NIR) luminescence^16^ or short decay lifetimes due to thermally activated delayed fluorescence (TADF).^17^ Selected Pt(II) complexes display strong Pt···Pt aggregation, leading to efficient near-infrared (NIR) photo- and electroluminescence.^18−22^ Therefore, effective strategies for increasing the solubility of the said complexes are needed but such that do not completely restrict Pt···Pt contacts required for their NIR luminescence in the solid state.

Processing emissive materials from a solution poses significant advantages in terms of their use in low-cost or large-area applications. For example, many features of potential commercial applications can be produced with inkjet, roll-to-roll printing, or slot dye coating.^23,24^ In this case, the emitter molecule must be highly soluble in solvents that would pose low environmental as well as health and safety hazards. For example, the use of popular chlorinated solvents, such as chloroform, chlorobenzene, or dichlorobenzene, must be avoided. Instead, halogen-free aromatic solvents, such as toluene or xylenes, can be used—ideally these can also be replaced with an appropriate green solvent.

Typically, white-light OLEDs or WOLEDs require two emitters, i.e., blue and yellow, to obtain white electroluminescence.^25^ This can be achieved by mixing the two or more luminophores in an emissive layer in a specific proportion or through the use of tandem and multilayer structures.^26,27^ A rather more favorable possibility is the use of a single dopant that may give rise to the two luminescent bands, which combined together may result in white electroluminescence. Single-dopant WOLEDs are significantly simpler, eliminating multiple variables that affect the optimal design of multicomponent structures. In this respect, excimer-forming platinum(II) complexes are a profound example of a luminophore where the control of the emitter concentration allows for fine-tuning of the EL color among sky blue, white, and yellow-orange.^14^

**Scheme 1:**
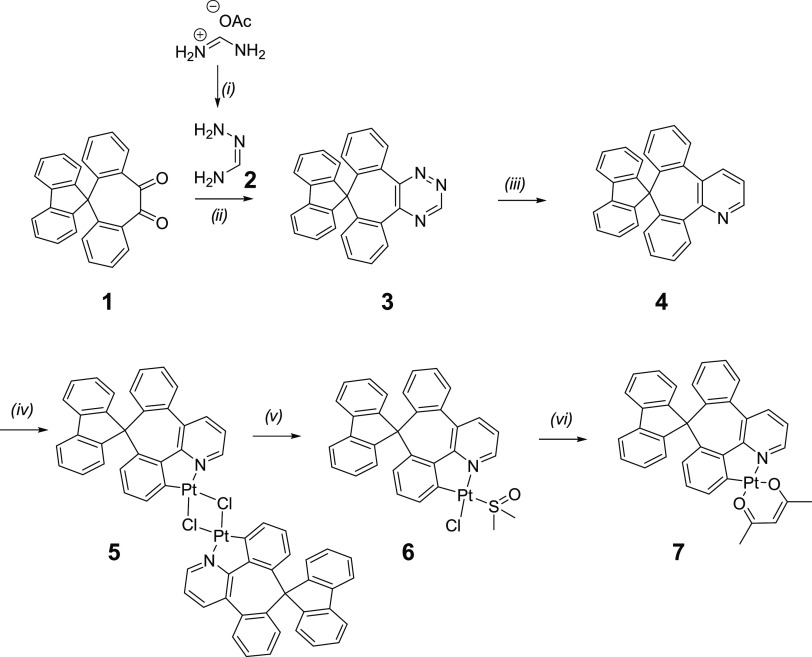
Synthesis of the Cyclometallating Ligand**4** and Its Pt(II) Complex**7** Reaction conditions: (i) hydrazine hydrate, RT, MeOH, 2 min, *in situ*; (ii) 1,4-dioxane/DMF, RT, 24 h, 34%; (iii) 2,5-norbornadiene, toluene, sealed reactor, 185 °C, 20 h, 65%; (iv) K_2_PtCl_4_, AcOH, reflux, 24 h; (v) DMSO, 130 °C, 30 min; (vi) Na(acac), acetone, reflux, 72 h, 17% (overall for steps iv–vi).

Considering the various types of chelating ligands in platinum(II) complexes,^1,28−32^ we have decided to use a phenylpyridine (ppy)-type C,N ligand with the coordination of the central ion completed with acetylacetonate (acac) following our earlier work.^33^ Diketone **1** (Scheme 1) was previously used to derive pyrazine-type cyclometallating ligands.^34^ Keeping the above factors in mind, we decided to use **1** as a basis to construct ligand **4** featuring the spiro-fluorene unit. To the best of our knowledge, the pyridine-type ligand **4** has not been previously reported. We therefore decided to prepare it and to investigate its coordinating behavior and thus obtain the platinum(II) complex **7**.

## Synthesis

In our synthesis, we used 1,2,4-triazine derivative **3** as a key intermediate. The 1,2,4-triazine methodology is a versatile tool that we and others previously used to access various polypyridine-type ligands.^7^ The diketone **1** was reacted with generated in situ formamidrazone **2** to give 1,2,4-triazine **3** in 34% yield. 1,2,4-Triazines are well-known electron-deficient dienes that participate in inverse electron demand Diels–Alder reactions with electron-rich dienophiles. To prepare unsubstituted pyridine, the triazine **3** was reacted with an excess of 2,5-norbornadiene to give the desired cyclometallating ligand **4** in 65% yield. The reaction required a high temperature of 185 °C. To avoid high-boiling-point solvents, which are difficult to remove, the reaction was carried out in toluene in a sealed pressure tube. It should be noted that the 1,2,4-triazine method allows functionalization of the pyridine ring by using a variety of electron-rich dienophiles, providing a tool for further tuning of the physical and emissive properties of the complexes. The ligand **3** was then used to prepare the target complex **7** in a well-known three-step procedure. First, the ligand was heated under reflux in acetic acid with potassium tetrachloroplatinate to give the dichloro-bridged intermediate **5**, which was then cleaved by heating with DMSO to give the DMSO complex **6**, which upon reacting with sodium acetylacetonate in refluxing acetone gave the desired product **7**. The overall yield of **3**–**7** was 17%.

## Photophysics

### Solution State

**Figure 1:**
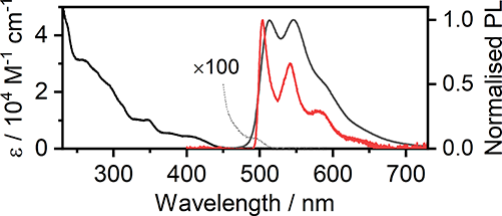
Absorption and PL spectra (black and dark-gray line, respectively) of **7** recorded in CH_2_Cl_2_ at RT as well as PL spectrum in 2MeTHF glass (red line) recorded at 77 K (*c* = 10^–5^ M).

**Figure 2:**
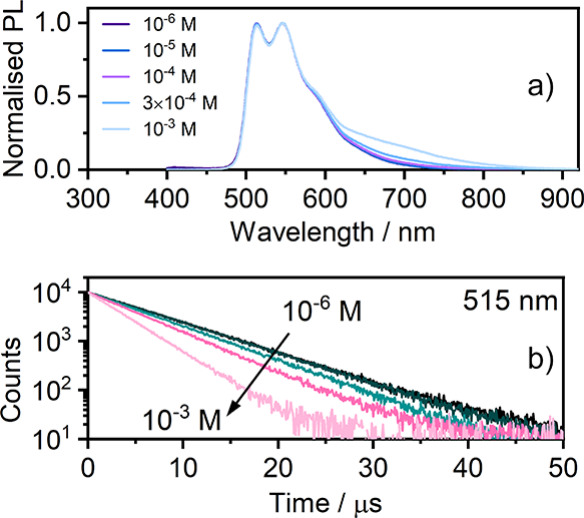
(a) PL spectra in CH_2_Cl_2_ recorded at concentrations from 10^–6^ to 10^–3^ M. (b) PL decay traces recorded at λ_col_ = 515 nm for concentrations of **7** from 10^–6^ to 10^–3^ M. Note that the shortening of phosphorescence lifetime in (b) is attributed to self-quenching, which gives rise to broadband excimer ^3^MMLCT emission visible in (a) the 600–800 nm range.

The absorption spectrum (Figure 1) of **7** in CH_2_Cl_2_ is rather typical of other platinum(II) complexes, with notable maxima at λ_abs_ = 260 nm (ε = 31 300 M^–1^ cm^–1^) and λ_abs_ = 345 nm (ε = 10 100 M^–1^ cm^–1^) and an absorption shoulder at λ_abs_ ∼ 405 nm (ε ∼ 4200 M^–1^ cm^–1^). Furthermore, we also observe an extremely weak absorption band at λ_abs_ = 493 nm (ε = 40 M^–1^ cm^–1^), attributed to a spin-forbidden *S*_0_ → *T*_1_ transition based on its overlap with the onset of the phosphorescence spectrum. Using the Strickler and Berg method,^35^ we estimate the radiative rate of the *T*_1_ → *S*_0_ transition, which is comparable to that obtained directly (listed below), *k*_r_ = 3 × 10^4^ s^–1^. This further confirms our attribution of the λ_abs_ = 493 nm band to an *S*_0_ → *T*_1_ transition. Complex **7** displays a vibronically resolved PL spectrum at RT (λ_PL_ = 514 nm, compared to λ_PL_ = 485 nm in [Pt(ppy)(acac)]^36^), with the vibronic structure even more evident at 77 K in 2MeTHF glass, with the (0,0) transition at 504 nm and extending out to 640 nm with a progression of ∼1400 cm^–1^ (∼170 meV). The complex is readily soluble in a variety of solvents and may be brought to a very high concentration. We study the complex in CH_2_Cl_2_ in the concentration range from 10^–6^ to 10^–3^ M (Figure 2), which reveals a fair to low propensity (quenching constant 1.5 × 10^8^ M^–1^ s^–1^, ∼30× lower than that reported for the archetypal excimer-forming Pt(bpyb)Cl^37^) to form low-energy excited states typically assigned to excimers. Thus, we attribute the reduction of the luminescence lifetimes visible in Figure 2b to self-quenching due to intermolecular collisions leading to the formation of bimolecular MMLCT excited states. This behavior is fully in line with that of other platinum(II) complexes.^3,38−40^ These findings indicate that the introduction of spiro-linked fluorene significantly reduces the effective intermolecular interactions that lead to aggregation or excimer formation in solution. The newly formed excimer displays a broadband PL with λ_PL_ = 682 nm (Figure S15). The overall contribution of the excimer PL is very low, even at 10^–3^ M, and therefore, the decay traces recorded at λ_col_ = 515 nm and at λ_col_ = 750 nm appear nearly identical. However, the decay at λ_col_ = 750 nm subtly lags behind the λ_col_ = 515 nm, which is a feature usually observed for excimer formation in platinum(II) complexes in solution (Figures S13 and S14). This is an expected behavior, which we have discussed in our earlier works.^37,41^ To further confirm that the observed long-wavelength PL band originates from excimers and not from ground-state aggregates, we record an absorption spectrum of a highly concentrated solution, *c* = 10^–3^ M (Figure S10). The absorption spectrum in this case is identical to that recorded at *c* = 10^–5^ M, indicating that no significant quantities of aggregates are present. The decay lifetime of **7** at RT in CH_2_Cl_2_ at *c* → 0 is estimated at τ = 7.1 μs. The lifetime rises to τ = 10.6 μs in the 2MeTHF glass at 77 K (*c* = 10^–5^ M). **7** displays a photoluminescence quantum yield in a solution of Φ_PL_ = 0.41 (higher than in [Pt(ppy)(acac)], where Φ_PL_ = 0.20^36^) and a triplet radiative rate of *k*_r_ = 6.0 × 10^4^ s^–1^ (virtually identical to that presented by [Pt(ppy)(acac)]^36^), very similar to that obtained using the Strickler and Berg method (see above).

### Solid State

**Figure 3:**
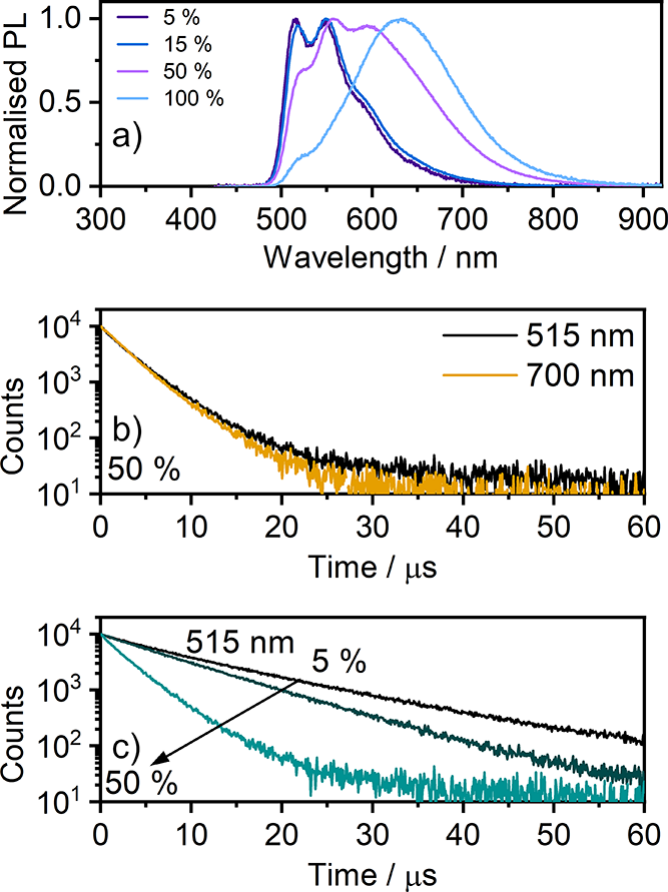
(a) PL spectra in the film of **7** doped into the PVK:PBD blend (5–50%) and the neat film. (b) PL decay traces recorded at λ_col_ = 515 nm and λ_col_ = 700 nm. (c) PL decay traces recorded at λ_col_ = 515 nm for concentrations of **7** in the PVK:PBD film from 5 to 50%.

We studied the behavior of **7** in a model OLED host, a blend of PVK {poly(*n*-vinylcarbazole)} and PBD {2-(4-biphenyl)-5-(4-*tert*-butylphenyl)-1,3,4-oxadiazole} (Figure 3). **7** displays a similar behavior in films to solution with a clearly resolved unimolecular PL, identical to that recorded in CH_2_Cl_2_. It also forms a broad and low-energy PL band (λ_PL_ = 636 nm). We assign this emission to dimers based on our previous studies.^15,37^ This assignment is in line with the behavior of the PL spectra in the film, where the intensity of the (0,0) component is reduced upon increased concentration due to a weak dimer absorption. A similar effect is not observed in a solution, where excimers dominate. Usually, excimers/dimers of a given Pt(II) complex are identical or at least very similar in solution and in film. This is due to the negligible effect of the environment on the excited-state energy of the bimolecular species. In fact, aggregate emission in a film tends to be more red-shifted due to the involvement of larger aggregates than dimers.^15,37^ In the case of **7**, we observe a more blue-shifted PL in the film with respect to the solution (Figure S15). This unusual behavior of the bimolecular emission suggests that the solvent may be stabilizing the excimer or that the solid-state dimer is more rigid (and hence this is a rigidochromic effect). We explore the bimolecular excited state of **7** in the computational section below.

**7** decays with an average lifetime (based on a biexponential fit) of τ_av_ = 13.0 μs and a Φ_PL_ = 0.39 in a 5% loaded film; a similar lifetime is recorded in a frozen solution. This highlights the similarity between the highly rigid solid phase at RT and a frozen solvent glass at 77 K. The decay lifetime of the 515 nm band shortens to only τ = 3.6 μs in the 50% loaded film (Φ_PL_ = 0.30) and further to τ = 1.3 μs in the 100% film (Φ_PL_ = 0.14) for collection at 700 nm in the last case. An expected behavior of the unimolecular (monomer) and the longer-wavelength (aggregate) bands in platinum(II) complexes in films is such that the former always decays with a longer lifetime than the latter.^37,42^ This is because the aggregate and monomer bands originate from distinct species existing in the ground state (i.e., aggregates formed from monomers) and decay naturally according to their respective radiative and nonradiative rates. In the case of **7** however, we observe identical decay lifetimes for collection at 515 and 700 nm for the 15 and 50% loaded films. This suggests that the species are in fact in the form of an equilibrium where one species convert into another in a time scale much shorter than the luminescent decay. Given the reduced molecular mobility in the film with respect to the solution, it is unlikely for the luminophore to significantly migrate within the given time scale. In this case, it is more likely for the molecules to oscillate around their centers of mass. We speculate that the behavior of the monomer and aggregate bands originates from loosely bound dimers MM, which can oscillate between local excitation on one of the two units M + M* and a bimolecular excited-state MM*. In other words, **7** appears to behave in the film as if the molecule formed typical excimers rather than dimers. Given however the low mobility of molecules in solid films, the most likely scenario is that they are already preorganized into structures similar to dimers, yet they are loose enough to be able to form bimolecular excited states, but also, then, to dissociate into M + M* as it normally happens in a solution. This behavior is highly unusual. Our assessment is further confirmed by the absorption spectra of **7** recorded in the film (Figure S16) as they show virtually the same absorption profile in a dilute CH_2_Cl_2_ solution and in a neat film, suggesting lack of formation of “conventional” aggregates. The molecules in the film behave as if they were isolated.

### Calculations

We employ density functional theory (DFT) and time-dependent DFT (TD-DFT) using Orca^43^ software to gain an in-depth understanding of the luminescent behavior of the monomeric complex **7** and its bimolecular excited state. Ground-state (*S*_0_) and triplet excited-state (*T*_1_) geometries were optimized at the B3LYP^44,45^/def2-TZVP^46^/CPCM(CH_2_Cl_2_) level of theory. Phosphorescent radiative rates were obtained with the quasi-degenerate perturbation theory (QDPT)^47,48^ using zeroth-order regular approximation (ZORA)^49,50^—corrected def2-TZVP basis sets^46^ for light atoms and a segmented all-electron relativistically contracted (SARC) def2-TZVP basis set for Pt.

**Figure 4:**
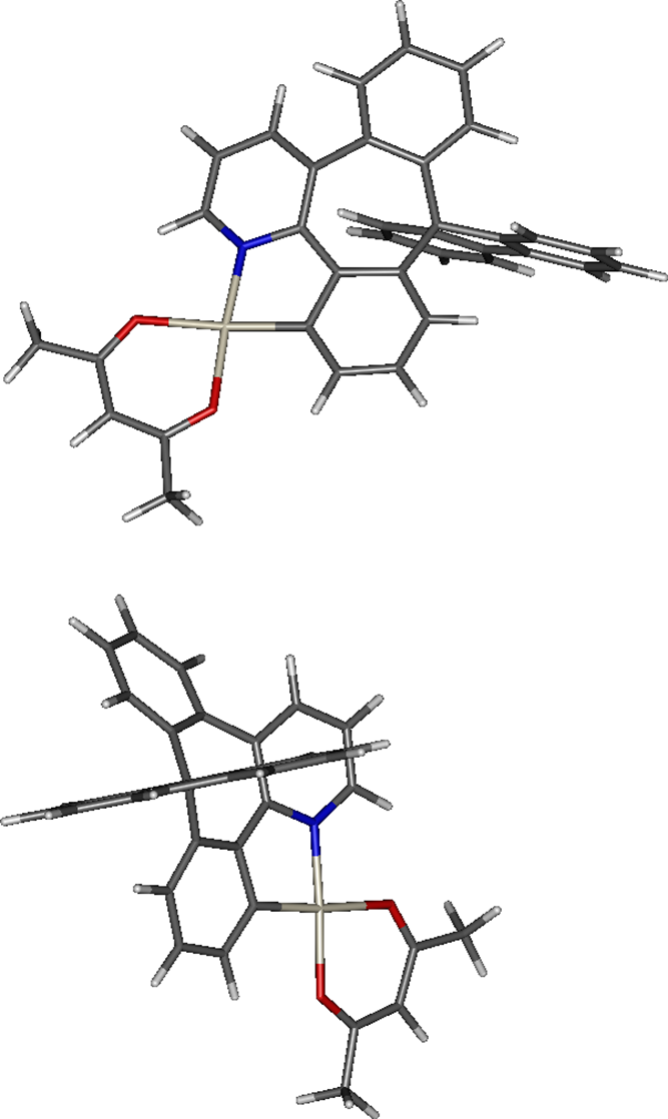
Top and bottom: Optimized *T*_1_ geometry of **7** shown from two different viewing perspectives.

**Figure 5:**
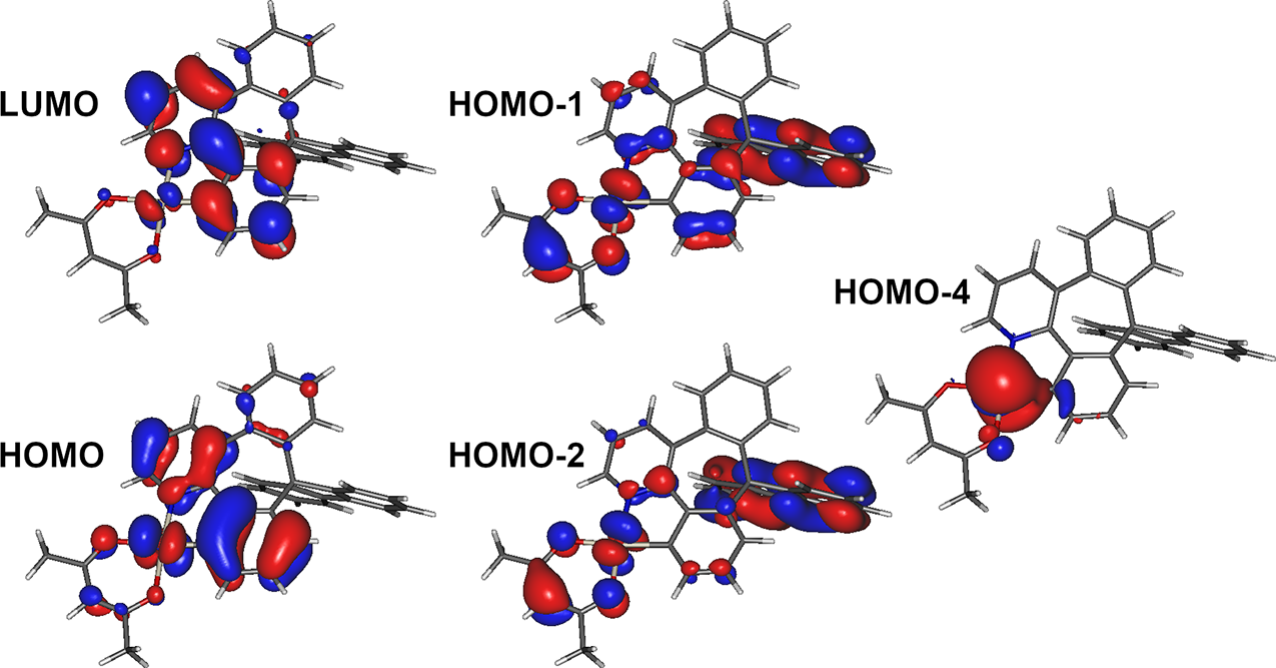
Iso surfaces of the molecular orbitals relevant to the *T*_1_ and *S*_1_–*S*_4_ excited states.

The molecule adapts a bent conformation of the *CN* ligand around the seven-membered ring with the spiro-linked fluorene unit, similar in *S*_0_ and *T*_1_ (Figure 4). We note that the experimental photoluminescence of **7**, λ_PL_ = 514 nm, is visibly red-shifted with respect to that of related complexes, which display λ_PL_ < 500 nm,^13,36^ especially in cases where the phenyl unit attached to the pyridine fragment is not connected to the *C*-coordinating part of the ligand and can rotate freely.^33^ In the case of **7**, the said phenyl unit is part planarized by the nonconjugating bridge to the *C*-coordinating fragment. We indeed observe that the noncoordinating third phenyl unit is to a small extent involved in the highest occupied molecular orbital (HOMO) and the lowest unoccupied molecular orbital (LUMO) at the *T*_1_-optimized geometry (Figure 5). However, otherwise, its role is minimal and the λ_PL_ of **7** remains still relatively close to that of the related complexes with phenylpyridine chelating ligands. The fluorene unit is perpendicular to the *XY* plane set out by the *CN* ligand, but also displaced outward of the structure along the *Z* axis. As a unit not conjugated with the rest of the *CN* ligand, the fluorene moiety was not initially expected to take part in the lowest excited states. It does not contribute to HOMO or LUMO, but it shows a clear contribution to HOMO-1 and HOMO-2, likely due to its mild electron-donating capability (Figure 5).

**7** displays a small calculated zero-field splitting (ZFS = 18.4 cm^–1^), which is the energy difference between the split sublevels of the *T*_1_ state. The calculated radiative rate of *k*_r_ = 9 × 10^4^ s^–1^, in full agreement with the experimental result. The ZFS value is consistent with the mixed ^3^LC (ligand centered)–^3^MLCT character of the emissive excited state^51^ and similar to the experimental value reported for the archetypal [Pt(ppy)(acac)], ZFS = 11.5 cm^–1^.^36,51^ The calculated energy of the *S*_0_ → *T*_1_ transition, 2.71 eV (457 nm), is in line with that recorded experimentally from the absorption spectrum. The phosphorescent properties of the complex originate from the “borrowing” of the singlet radiative rate to the *T*_1_.^52^ For example, a modest *S*_1_–*T*_1_ spin–orbit coupling matrix element, SOCME = 23 cm^–1^, is related to the similarity of the two states, both composed of >0.9 HOMO → LUMO excitation. Stronger coupling occurs for *S*_2_*–T*_1_, SOCME = 597 cm^–1^, and *S*_3_–*T*_1_, SOCME = 270 cm^–1^, pairs due to the involvement of a different *d* orbital of the metal center in *S*_2_ (HOMO-1 → LUMO, 0.78) and *S*_3_ (HOMO-2 → LUMO, 0.80; HOMO-1 → LUMO, 0.13). An even stronger coupling is displayed for the *S*_4_–*T*_1_ pair, SOCME = 1250 cm^–1^, where the *S*_4_ (HOMO-4 → LUMO, 0.78) involves nearly exclusively the *d*_z^2^_ orbital of the metal center. Diagrams representing the lowest singlet and triplet excited states can be found in the SI, Figure S19.

**Figure 6:**
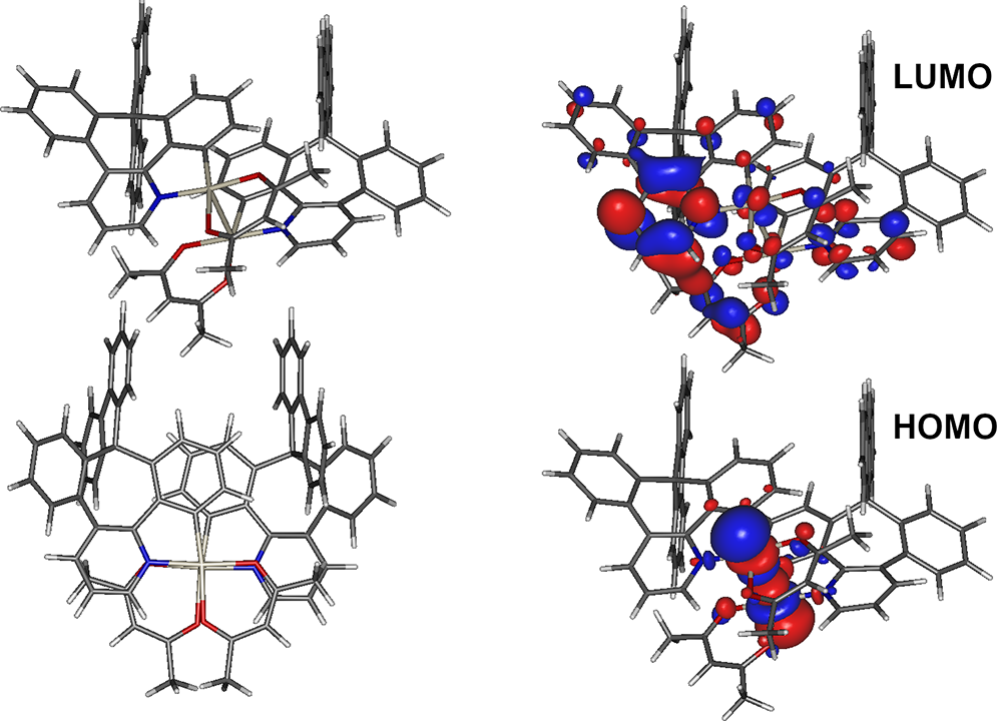
Excited-state dimer of **7**: (left) proposed optimized *T*_1_ structure; (right) frontier molecular orbital iso surfaces.

In order to study the properties of the **7** excimer/dimer, we constructed a model T_1_ state dimer following the approach used successfully by us earlier.^15,37^ The initial geometry comprised two molecules with a Pt···Pt distance of ∼3 Å and with the bulky fluorene fragments facing outward. The geometry was then optimized at the BP86/def2-svp level of theory, while the single-point energy calculation at the optimized *T*_1_ geometry was performed with B3LYP/def2-svp/CPCM(CH_2_Cl_2_). The structure of the excited-state dimer presented in Figure 6 displays the only minimal geometry obtained. For example, Pt(NCN)-X-type complexes usually display two minima: head-to-tail and head-to-head structures, with the former being the one most likely present in experimental systems.^37^ In the case of **7**, the two molecules are rotated one to another around the Pt···Pt axis by about 90°, with all of the other conformers prohibited due to the interaction between the fluorene unit and methyl groups of the acetylacetonate (acac) ancillary ligand. The dimer geometry displays a Pt···Pt distance of 2.82 Å, and the fluorene units are pointing inward of the structure. The coordination plane around the Pt centers is deformed from planarity, with both the acac and phenylpyridine ligands tilted slightly outward of the structure, but themselves remaining planar. The emissive triplet state, *T*_1_ = 1.63 eV (760 nm), is of a HOMO → LUMO nature. The HOMO is localized on the two metal centers with a clear contribution of their *d*_z^2^_ orbitals pointing toward each other, while the LUMO is distributed over the π-conjugated CN ligand, mostly on its pyridine fragment, forming an ^3^MMLCT excited state.

### Electrochemistry

**Figure 7:**
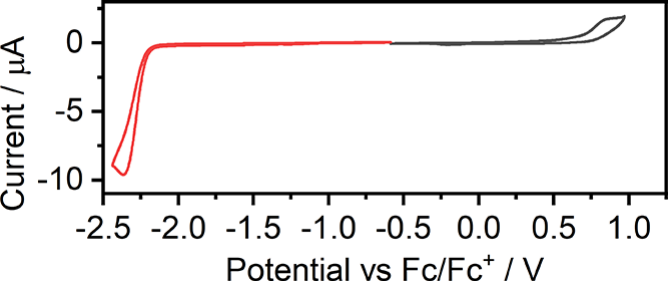
Electrochemical redox processes were recorded for **7** with cyclic voltammetry at a scan rate of 50 mV s^–1^. Red, reduction cycle; black, oxidation cycle.

Cyclic voltammetry of **7** reveals a rather typical picture of a platinum(II) complex electrochemistry where the ligand displays only a mildly electron-deficient nature (Figure 7). In such a case, both the oxidation and reduction cycles are irreversible. This behavior is in line with the electrochemical properties of analogous complexes of acac and ppy-like ligands, which also reduce irreversibly.^33^ For example, reduction is often reversible in complexes containing a more electron-deficient pyrimidine,^53^ but oxidation nearly always remains irreversible, unless the oxidated state is stabilized by the chelating ligand.^54^ Complex **7** displays electrochemically derived HOMO and LUMO energies at −5.77 and −2.89 eV, respectively (Table S1).

### Electroluminescence

**Figure 8:**
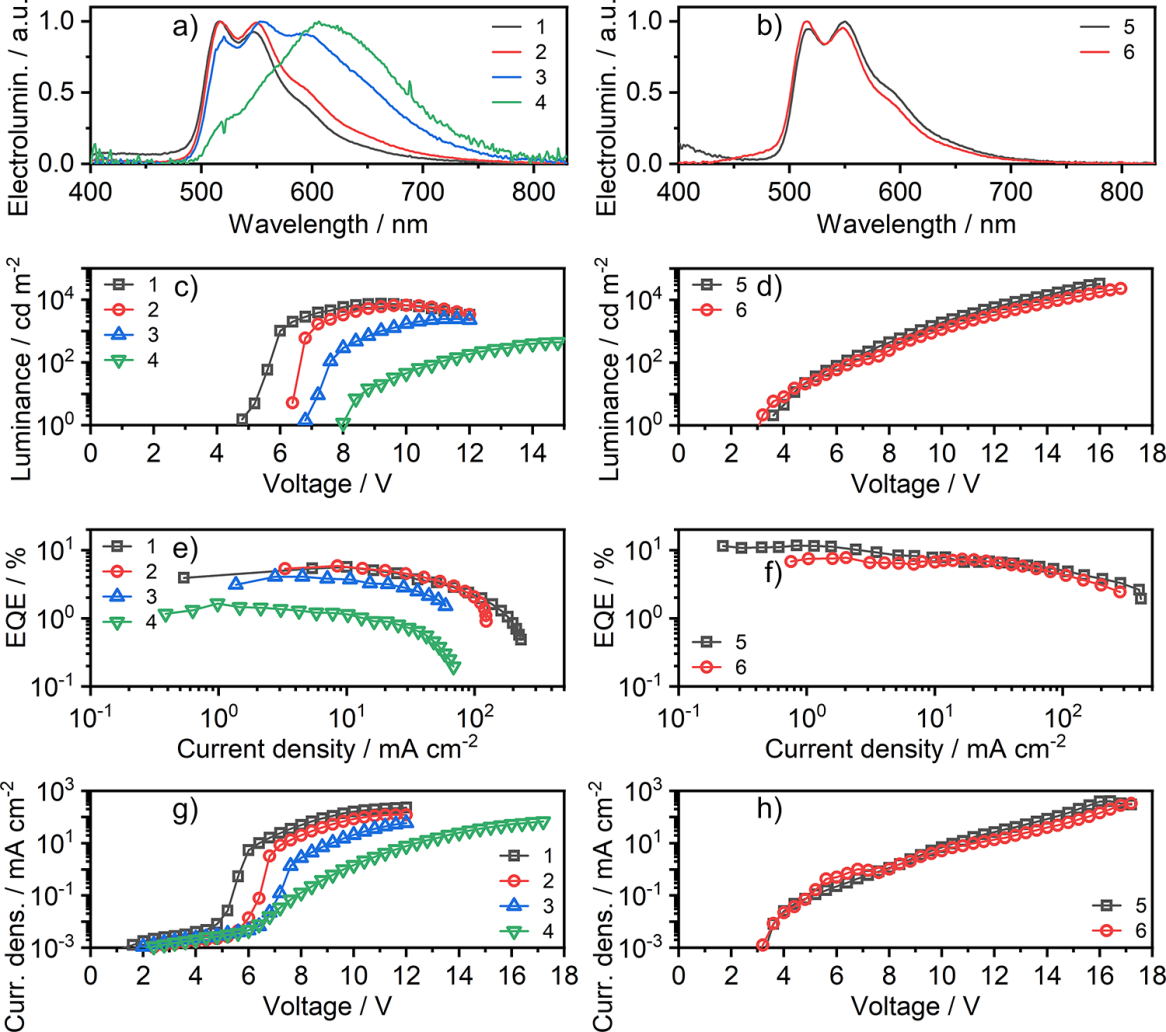
Characteristics of solution-processed OLED devices 1–4 and vacuum-deposited devices 5 and 6: (a, b) electroluminescence spectra; (c, d) luminance vs applied voltage; (e, f) external quantum efficiency (EQE) vs current density; and (g, h) current density vs applied voltage.

We produced prototype OLEDs to demonstrate the potential application of **7** in optoelectronic devices (Figure 8, Table 1). First, owing to the profound solubility of **7** in toluene, we have produced solution-processed OLEDs 1–4 with a variable emitter load, from 5 to 100%, using a popular PVK:PBD host blend. Great solubility of the platinum(II) complex in toluene allows for the use of a more balanced structure involving a PVKH hole transport and an electron blocking layer,^55^ in comparison with structures where the emissive layer is placed directly on top of PEDOT:PSS. This structure allows for a relatively low turn-on voltage of ∼5–7 V and luminance of up to 7400 cd m^–2^. Progressively increasing the emitter load from 5 to 100% in the emissive layer results in a gradual increase in the contribution of the broadband and a longer-wavelength emission band, up to its dominance in the host-free OLED. The maximum external quantum efficiency (EQE) of the resultant OLEDs 1–4 follows the PLQY of the EML, with the maximum value of 5.9% for the 15% loaded EML and the minimum value of 1.7% for the nondoped device. A close comparison of the PLQY and EQE values suggests that OLEDs 1–4 may not be fully optimized. Therefore, we fabricated fully vacuum-deposited OLEDs, which can be optimized more easily. Thus, the obtained OLEDs 5 and 6 featuring different electron transport components display higher EQEs of 11.8 and 7.5%, respectively, and high luminance of up to 32 500 cd m^–2^ with low turn-on voltage around ∼3–3.5 V. The design of OLEDs 5 and 6 follows previous successful applications of similar architectures to thermally activated delayed fluorescence (TADF) emitters.^56,57^ These structures are based on a two-component blend host mCP:X, where X = T2T for device 5 and X = PO-T2T for device 6 with the X = T2T or PO-T2T, respectively, serving also as the hole blocking layer. Detailed architectures of devices 1–6 can be found in the SI, Table S5.

Although **7** does not display white electroluminescence due to the insufficiently blue-shifted monomer PL, the behavior of the complex highlights the possibility of using a similar molecular design in WOLEDs. In our case, the small energy difference between the uni- and bimolecular emissions favors applications in WOLEDs. This is because the lower energy ^3^MMLCT band is relatively blue shifted, thus most of it remains in the visible region. In consequence, the bimolecular band contributes more to the visible spectrum as the fraction falling into the near-infrared (>700 nm) region remains negligible. A further modification of complex **7** could include decoration of the phenylpyridine fragment of the CN ligand with electron-withdrawing groups to shift the monomer emission further to blue.^58^

**Table 1:** Characteristics of the OLED Devices 1–6

	Dev 1	Dev 2	Dev 3	Dev 4	Dev 5	Dev 6
emitter load, %	5	15	50	100	10	10
*V*_ON_/*V*^a^	4.8	6.4	6.8	8.0	3.5	3.1
*L*_max_/cd m^–2^^b^	7400	6700	2300	400	32500	23300
λ_EL_/nm^c^	517, 547, 590sh	517, 550, 594sh	519, 555, 595	522sh, 609	517, 550, 594sh	516, 548, 594sh
CIE 1931 (*x*, *y*)^d^	(0.34, 0.56)	(0.37, 0.58)	(0.45, 0.52)	(0.52, 0.46)	(0.36, 0.56)	(0.34, 0.58)
CE_max_/cd A^–1^^e^	19.8	19.9	10.5	3.8	38.9	25.5
EQE_max_/%^f^	5.7	5.9	4.1	1.7	11.8	7.5

a Turn-on voltage at 1 cd m^–2.^

b Maximum luminance.

c Electroluminescence maxima.

d Color coordinates of the electroluminescence spectrum as defined in the International Commission on Illumination color space CIE 1931.

e Maximum current efficiency.

f Maximum external quantum efficiency.

## Conclusions

The 1,2,4-triazine methodology was used to access a novel cyclometalating ligand that contains a spiro-fluorene unit. The ability of the ligand to form cyclometalated complexes was proven by the synthesis of complex **7**. Other metal ions such as Ir(III) can potentially be used to provide access to a great variety of structures. The nonplanar geometry of the ligand leads to high solubility of the complex in toluene and other solvents and effectively suppresses the excimer formation in solution. However, in the solid state, aggregation-type emission is observed for a 100% layer.

We observe an unusual behavior of the excimer/dimer ^3^MMLCT photoluminescence band formed by **7** at a high concentration in the film and in the solution. First, the long-wavelength band is more red-shifted in the solution than in the film, while they should rather be identical, indicating that most likely the “rigid” environment of the solid film stabilizes the excimer, while its stability is lower in the solution (i.e., this is a rigidochromic effect). Second, the ^3^MMLCT band displays similar kinetics in the film and in the solution, which is fundamentally surprising since the mechanisms underpinning formation of this PL band are different in the two media. We suggest that **7** displays an excimer-like behavior in the film, where the dimeric excited-state MM* and the monomer–excited monomer pair M + M* are in an equilibrium, but without the associated relative displacement of the two units. This behavior is unexpected since typically we observe the aggregate ^3^MMLCT and the monomer ^3^MLCT PL bands to decay independently, reflecting an aggregation-type scenario.

Finally, we use the novel complex **7** as the emitter in solution-processed and vacuum-deposited OLEDs. Thanks to the profound solubility of **7** in toluene, we were able to obtain solution-processed emissive layers with 5–100% dopant content and a maximum luminance of 7400 cd m^–2^ and ∼6% EQE for the 5%-doped device. The fully vacuum-deposited OLEDs reached 32 500 cd m^–2^ and 11.8% EQE.

## Supporting Information Available

Supporting ^1^H and ^13^C NMR spectra; experimental details; supporting electrochemistry; photophysics; computational; and OLED characterization results (PDF)
Calculated_geometries (ZIP)

## Notes

A version of this manuscript has been submitted to the ChemRxiv: 10.26434/chemrxiv-2023-fxzxw.^59^
