# Supplementary material for: Unusual Excimer/Dimer Behavior of a Highly Soluble C,N Platinum(II) Complex with a Spiro-Fluorene Motif
Source: Inorg Chem. 2023 Oct 31;62(45):18465–73. doi: 10.1021/acs.inorgchem.3c02667 (PMC10647119; doi:10.1021/acs.inorgchem.3c02667)
Supplement: Supplementary file 1 — ic3c02667_si_001.pdf [file ic3c02667_si_001.pdf]

Supporting Information

Unusual excimer/dimer behaviour of a highly soluble C,N platinum(II) complex with a spiro-fluorene motif

Piotr Pander,[a],[b]* Andrey V. Zaytsev,[c] Larissa Gomes Franca,[d],[e] Fernando B. Dias[d]* and Valery N. Kozhevnikov[c]*

[a] *Faculty of Chemistry, Silesian University of Technology, Strzody 9, 44-100 Gliwice, Poland*
E-mail: piotr.pander@polsl.pl
[b] *Centre for Organic and Nanohybrid Electronics, Silesian University of Technology, Konarskiego 22B, 44-100 Gliwice, Poland*
[c] *Department of Applied Sciences, Faculty of Health and Life Sciences, Northumbria University, Newcastle Upon Tyne, Tyne and Wear NE1 8ST, UK*
E-mail: valery.kozhevnikov@northumbria.ac.uk
[d] *Department of Physics, Durham University, Durham, South Road, DH1 3LE, UK*
E-mail: f.m.b.dias@durham.ac.uk
[e] *Department of Materials Science and Metallurgy, University of Cambridge, Cambridge CB3 0FS, UK*

Table of contents

1. General 2
2. Synthesis 5
3. NMR spectra 7
4. Photophysics 16
5. Electrochemical data 18
6. Quantum chemical calculations 18
7. Electroluminescent devices 19
8. References 21

1. General

Materials and Methods

All solvents and reagents were purchased from Sigma-Aldrich, Acros Organics or Alfa-Aesar and used without further purification unless otherwise specified. Reactions were monitored by TLC using silica gel with UV_{254} fluorescent indicator. NMR spectra were recorded on a JEOL ECS400FT Delta spectrometer (399.78 MHz for ^{1}H NMR, 100.53 MHz for ^{13}C NMR). Chemical shifts are reported in parts per million (ppm) relative to tetramethylsilane as internal standard. Coupling constants (J) are measured in hertz. Multiplets are reported as follows: b = broad, s = singlet, d = doublet, dd = double doublet, t = triplet, q = quartet, qu = quintet, m = multiplet, app d = apparent doublet, app t = apparent triplet.

The starting diketone derivative **1** was prepared as described previously.[1]

Calculations

We use density functional theory (DFT) and time-dependent DFT (TD-DFT) as well as the quasi-degenerate perturbation theory (QDPT)[2,3] with zeroth-order regular approximation (ZORA)[4,5] implemented in Orca 5.0.3[6,7] in order to gain an additional insight into the phosphorescent properties of **7**. Ground state (S_0) and triplet excited state (T_1) geometries were optimised at the B3LYP[8,9]/def2-TZVP[10]/CPCM(CH_2Cl_2) level of theory. Singlet and triplet radiative rates were calculated using ZORA-corrected def2-TZVP basis sets[10] for light atoms and a segmented all-electron relativistically contracted (SARC) def2-TZVP basis set for Pt. All molecular orbital (MO) iso surfaces were visualised using Gabedit 2.5.0.[11]
Geometry optimisations were performed at the B3LYP[8,9]/def2-TZVP[10] level of theory with RIJCOSX[12,13] approximation to accelerate calculations and def2/J[14] auxiliary basis set. Atom-pairwise dispersion correction with the Becke-Johnson damping scheme (D3BJ)[15,16] was included in the calculation. All geometries were verified to be true energy minima by a frequency calculation. All optimisations were performed with tight SCF and geometry convergence criteria. Excited state energy of TDDFT states was calculated using the resultant S_0 or T_1 geometry. In this case relativistically corrected triple-zeta basis sets with the zeroth-order regular approximation (ZORA)[4,5] were used: ZORA-def2-TZVP[10] with the SARC/J[17] auxiliary basis for all atoms except Pt for which a segmented all-electron relativistically contracted (SARC) SARC-ZORA-TZVP[17] basis set was used. Spin-orbit coupling (SOC) calculations were performed as implemented in the ORCA software. SOC matrix elements (SOCME) and SOC-corrected excitations (SOC states) were computed using the same settings as for the TDDFT states. In order to accelerate the calculations RIJCOSX[12,13] approximation was used in all cases and the RI-SOMF(1X) setting was used to accelerate SOC calculations.

Electrochemistry

Cyclic voltammetry was conducted using a three-electrode, one-compartment cell. All measurements were performed using 0.1 M Bu_4NBF_4 (99%, Sigma Aldrich, dried) solution in dichloromethane (ExtraDry AcroSeal®, Acros Organics). All solutions were purged with nitrogen prior to measurement and the measurement was conducted in a nitrogen atmosphere. Electrodes used in the experiment were: working (Pt disc d = 1 mm), counter (Pt wire), and reference (Ag/AgCl calibrated against ferrocene). All cyclic voltammetry measurements were performed at room temperature with a scan rate of 50 mV s^{-1}.

Ionization potential (IP) and electron affinity (EA) are obtained from onset redox potentials; these figures correspond to HOMO and LUMO values, respectively. The ionization potential is calculated from onset oxidation potential $IP = E_{ox}^{CV} + 5.1$ and the electron affinity is calculated from onset reduction potential $EA = E_{red}^{CV} + 5.1$.[18,19,20,21] An uncertainty of ±0.02 V is assumed for the electrochemical onset potentials.

Photophysics

Absorption spectra of solutions were recorded with UV-3600 double beam spectrophotometer (Shimadzu). Photoluminescence (PL) spectra of solutions and films were recorded using a QePro compact spectrometer (Ocean Optics) or a FluoroLog fluorescence spectrometer (Jobin Yvon). Time-resolved decays in film and solution used for determination of lifetimes were recorded with a Horiba DeltaFlex TCSPC system using a 330 nm SpectraLED or 405 nm DeltaDiode light sources. Temperature-dependent experiments were conducted using a liquid nitrogen cryostat VNF-100 (sample in flowing vapour, Janis Research) under nitrogen atmosphere, while measurements at room temperature were recorded under vacuum in the same cryostat. Solutions were degassed using five freeze-pump-thaw cycles. Thin films were deposited from chloroform or toluene solutions. The films were fabricated through spin-coating and dried under vacuum at room temperature.

Electroluminescent devices

OLEDs were fabricated by thermal evaporation or by spin-coating / evaporation hybrid method. We used pre-cleaned indium-tin-oxide (ITO) coated glass substrates with a sheet resistance of 20 Ω/sq and ITO thickness of 100 nm. The substrates were first washed with acetone and then sonicated in acetone and isopropanol, for 15 min each time. Substrates were dried with compressed air and transferred into an ozone-plasma generator for 6 min at full power. Thermally deposited layers were obtained using Kurt J. Lesker Spectros II deposition system at 10^{-6} mbar base pressure. All organic materials and aluminium were deposited at a rate of 1 Å s^{-1}. The LiF layer was deposited at a rate of 0.1–0.2 Å s^{-1}. Characterisation of OLED devices was conducted in a 10 inch integrating sphere (Labsphere) connected to a Source Measure Unit (SMU, Keithley) and coupled with a matrix spectrometer USB4000 (Ocean Optics). Further details are available in reference.[22] Devices of 4 × 2mm pixel size were fabricated.

Substances used for OLED fabrication have been purchased from suppliers indicated in parentheses: HAT-CN – dipyrazino[2,3-f:2',3'-h]quinoxaline-2,3,6,7,10,11-hexacarbonitrile (sublimed, LUMTEC); TSBPA – 4,4'-(diphenylsilanediyl)bis(N,N-diphenylaniline) (LUMTEC); mCP – 1,3-bis(carbazol-9-yl)benzene (sublimed, LUMTEC); T2T – 2,4,6-tris(biphenyl-3-yl)-1,3,5-triazine (sublimed, Ossila); PO-T2T – 2,4,6-Tris[3-(diphenylphosphinyl)phenyl]-1,3,5-triazine (LUMTEC); TPBi - 2,2',2"-(1,3,5-benzinetriyl)-tris(1-phenyl-1-*H*-benzimidazole) (LUMTEC); PBD – 2-(4-biphenyl)-5-(4-*tert*-butylphenyl)-1,3,4-oxadiazole (Sigma Aldrich); PVKH – poly(*N*-vinylcarbazole) (Sigma Aldrich, M = 10^6 Da); PVK – poly(9-vinylcarbazole) (M = 90 000 Da, Acros Organics); LiF (99.995%, Sigma Aldrich); Al pellets (99.9995%, Lesker).

Thermal evaporation. The fully thermally deposited OLEDs comprised hole injection layer: HAT-CN, hole transport layer: TSBPA, exciton blocking layer: mCP, hole blocking layer: PO-T2T or T2T, electron transport layer: TPBi, electron injection layer: LiF and cathode: Al. Optimised emissive layer comprised a blend host: mCP (hole transport component) and PO-T2T or T2T (electron transport component) for balanced carrier ratio and improved charge injection into the emitter.

Solution processing. Hole injection layer (PEDOT AI 4083, Ossila) was spin-coated and annealed on a hotplate at 120 ˚C for 15 min to give a 30 nm film. PVKH layer was spun from a

chloroform:chlorobenzene (95:5 v/v) solution (3 mg mL^{-1}) at 8000 RPM to yield a 10 nm layer. Consequently, the emissive layer was deposited from a toluene solution (10 mg mL^{-1}) at 2000 RPM to give a 30 nm layer. The dopant was dissolved in the solution of blend host in order to obtain final 5-50% (w/w) concentration in the emitting layer. All solutions were filtrated directly before use with a PVDF (organic solvents) and PES (PEDOT AI4083) syringe filter with a 0.45 μm pore size. The electron transport (TPBi) and electron injection (LiF) layers as well as cathode (Al) were thermally evaporated.

2. Synthesis

3

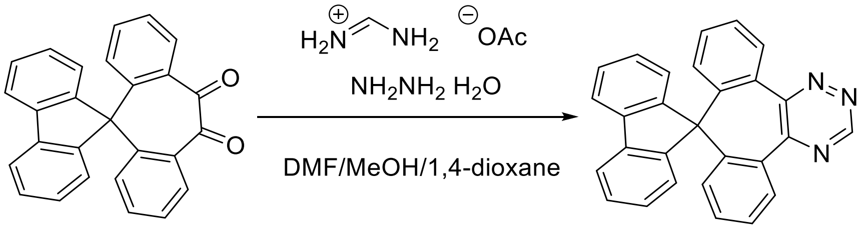

Hydrazine hydrate (90 mg, 87 µL, 1.79 mmol) was added to a solution of formamidine acetate (187 mg, 1.79 mmol) in methanol (8mL) and the reaction mixture was allowed to stir at room temperature for 2 mins. The reaction mixture was added to a solution of **1** (557 mg, 1.50 mmol) in a mixture of 1,4-dioxane (3.5 mL) and DMF (5 mL). The reaction mixture was stirred at room temperature overnight. Water (50 mL) was added and the organics were extracted with DCM (3 × 30 mL). The combined organic layers were washed with water (3 × 30 mL), dried over $MgSO_4$ and the volatiles were evaporated under reduced pressure. The residue was purified by flash column chromatography using pet. ether : ethyl acetate = 100:0 → 80:20 as an eluent to give **3** (198 mg, 34 %) as a yellow solid.

^{1}H NMR ($CDCl_3$, 400 MHz): δ 9.88 (s, 1H), 8.37 (ddd, *J* = 1.4, 6.9 and 7.8 Hz, 2H), 7.77 (d, *J* = 7.3 Hz, 2H), 7.48 – 7.42 (m, 2H), 7.38 (app.t, *J* = 7.3 Hz, 2H), 7.29 – 7.23 (m, 2H), 7.19 – 7.13 (m, 4H), 6.78 (d, *J* = 7.8 Hz, 2H).

^{13}C NMR ($CDCl_3$, 101 MHz): δ 159.1 (C), 156.3 (C), 155.9 (CH), 149.3 (C), 147.2 (C), 145.8 (C), 140.1 (C), 134.9 (C), 134.2 (C), 133.0 (CH), 132.6 (CH), 131.8 (CH), 130.6 (CH), 129.1 (CH), 128.9 (CH), 128.4 (CH), 128.3 (CH), 128.2 (CH), 127.5 (CH), 127.4 (CH), 120.8 (CH), 66.3 (C).

HRMS (m/z): (ESI) calc. for $C_{28}H_{18}N_3$ $[M+H]^+$: 396.14948; found: 396.15012

4

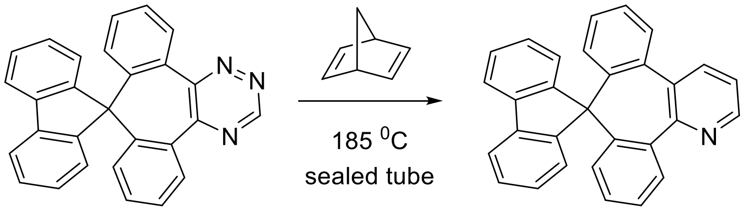

A solution of **3** (149 mg, 377 µmol) in 2,5-norbordiene (2 mL) was heated at 185 ^{0}C in a pressure tube for 20 h. The volatiles were evaporated and the residue was purified via flash column chromatography using pet. ether/DCM (100:0 → 0:100) as an eluent to give **4** (96 mg, 65%) as a light yellow solid.

^{1}H NMR ($CDCl_3$, 400 MHz): δ 8.93 (d, *J* = 4.1Hz, 1H), 8.23 (d, *J* = 7.3 Hz, 1H), 7.87 (d, *J* = 7.8 Hz, 2H), 7.60 – 7.49 (m, 6H), 7.39 (app.t, *J* = 7.3 Hz, 1H), 7.32 – 7.29 (m, 1H), 7.18 (d, *J* = 8.2 Hz, 2H), 7.14 – 7.06 (m, 4H), 6.65 (br s, 1H), 5.85 (br s, 1H).

^{13}C NMR ($CDCl_3$, 101 MHz): δ 156.7 (C), 149.1 (CH), 145.8 (C), 145.6 (C), 141.4 (C), 139.8 (CH), 139.7 (C), 138.0 (C), 136.9 (C), 132.5 (CH), 131.5 (CH), 131.3 (CH), 128.7 (CH), 128.6 (CH), 128.3 (CH), 128.1 (CH), 127.9 (CH), 127.8 (CH), 127.8 (CH) 126.4 (CH), 125.0 (CH), 122.8 (CH), 120.7 (CH), 120.1 (CH), 66.3 (C).

HRMS (m/z): (ESI) calc. for $C_{30}H_{20}N$ $[M+H]^+$: 394.15948; found: 394.15921.

7

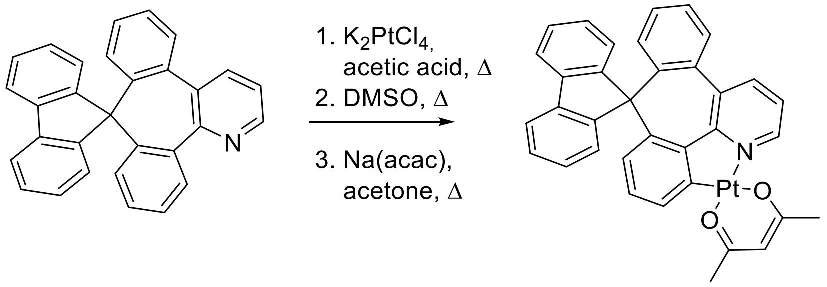

A reaction mixture containing **4** (291 mg, 0.74 mmol), K_2PtCl_4 (338 mg, 0.81 mmol) and acetic acid (150 mL) was heated under reflux, under Ar atmosphere for 24 h. Acetic acid was rotary evaporated under reduced pressure to a volume of approximately 10 mL. Water (30 mL) was added and the solid was filtered off and washed with water (30 mL). The solid was air-dried and used for the next step without further purification.

The above solid was heated in DMSO (3 mL) at 130°C under Ar atmosphere for 30 min. The reaction mixture was cooled to room temperature, diluted with water (50 mL) and the solid was filtered off washing with water. The dark-green solid was air-dried and used in the next step without further purification.

The solid from the previous step was dissolved in acetone (30 mL) and Na(acac) (963 mg, 7.89 mmol) was added. The reaction mixture was heated to reflux under Ar atmosphere for 3 days. The reaction mixture was cooled to room temperature and filtered through a plug of Celite washing with DCM. The solvents were removed under reduced pressure and the residue was purified via "dry flash" column chromatography using a mixture of pet. ether:DCM = 1:1 as an eluent to give **7** (86 mg (17% over three steps)) as an orange solid.

^{1}H NMR ($CDCl_3$, 400 MHz): δ 9.32 (d, *J* = 5.5 Hz, 1H), 8.02 (d, *J* = 7.8 Hz, 1H), 7.72 (d, *J* = 7.3 Hz, 2H), 7.62 (d, *J* = 6.4 Hz, 1H), 7.44 (d, *J* = 7.8 Hz, 1H), 7.35 – 7.29 (m, 4H), 7.23 (d, *J* = 7.8 Hz, 2H), 7.22 – 7.13 (m, 4H), 6.89 – 6.86 (m, 2H), 5.47 (s, 1H), 2.04 (s, 3H), 1.98 (s, 3H).

^{13}C NMR ($CDCl_3$, 101 MHz): δ 186.2 (C), 184.6 (C), 166.5 (C), 150.8 (C), 149.3 (C), 146.4 (CH), 145.3 (C), 144.6 (C), 144.0 (C), 143.8 (C), 143.0 (C), 141.8 (C), 140.4 (CH), 139.8 (C), 137.3 (C), 135.5 (C), 132.4 (CH), 129.9 (CH), 129.3 (C), 128.8 (CH), 128.6 (CH), 128.0 (CH), 127.9 (CH), 127.7 (CH), 127.2 (CH), 124.4 (CH), 121.1 (CH), 120.4 (CH), 102.7 (CH), 65.4 (C), 28.4 (CH_3), 27.3 (CH_3).

HRMS (m/z): ($ASAP^+$) calc. for $C_{35}H_{26}NO_2{}^{194}Pt$ $[M+H]^+$: 686.1590; found: 686.1605.

3. NMR spectra

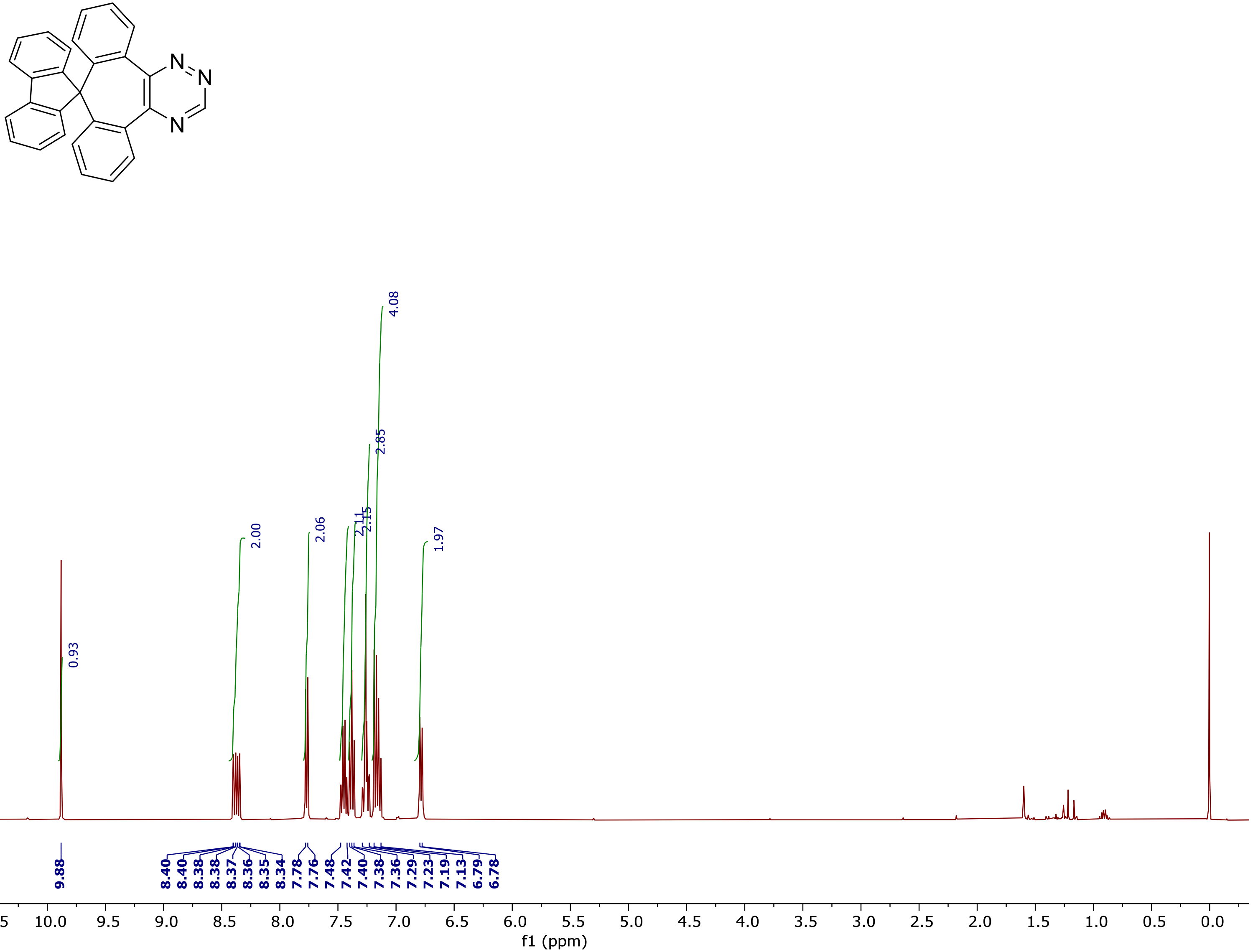

Figure S1. ^{1}H NMR spectrum of compound **3** (300 MHz, $CDCl_3$).

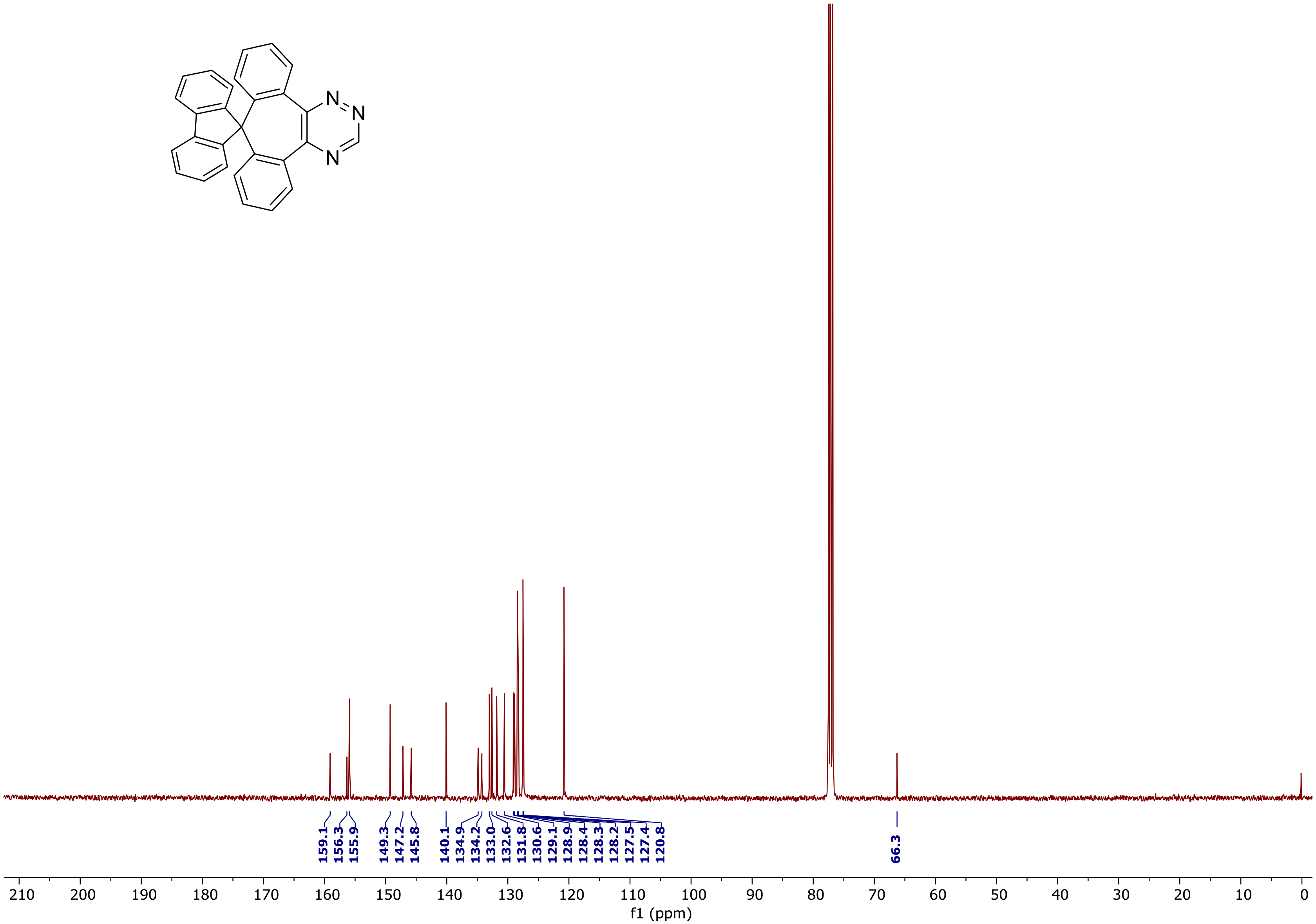

Figure S2. ^{13}C NMR spectrum of compound **3** (100.6 MHz, $CDCl_3$).

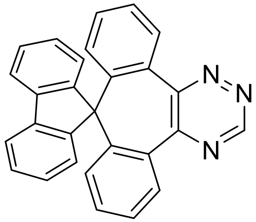

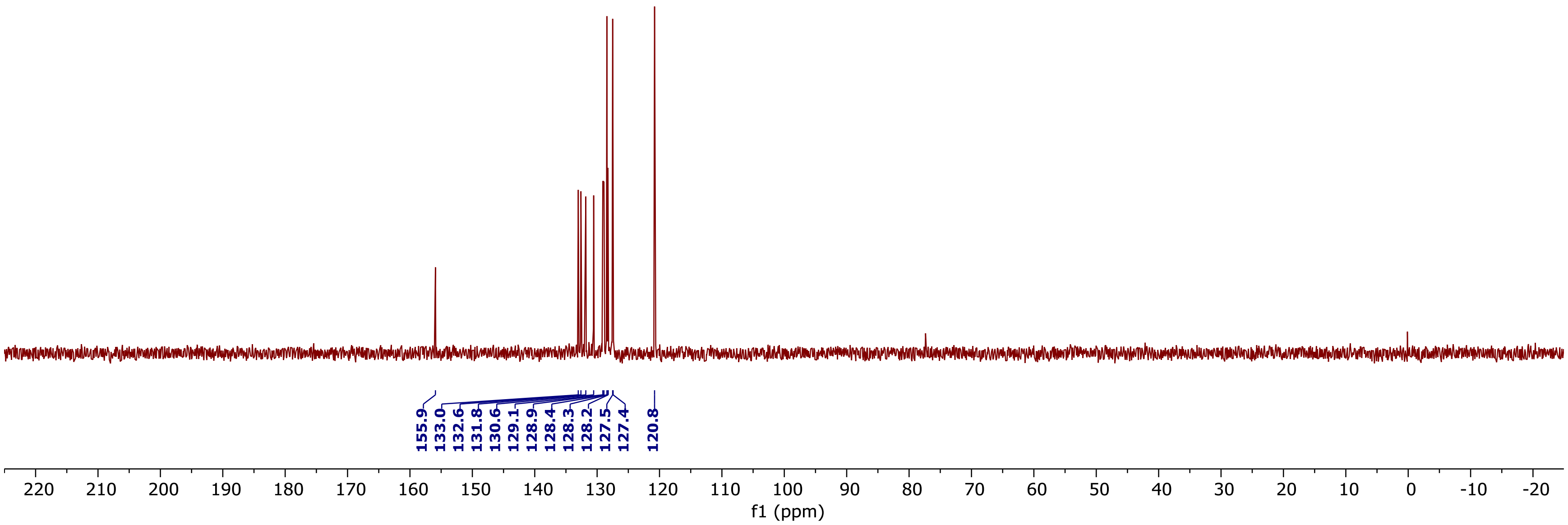

Figure S3. DEPT-135 ^{13}C NMR spectrum of compound **3** (100.6 MHz, $CDCl_3$).

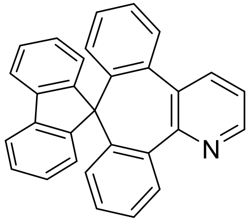

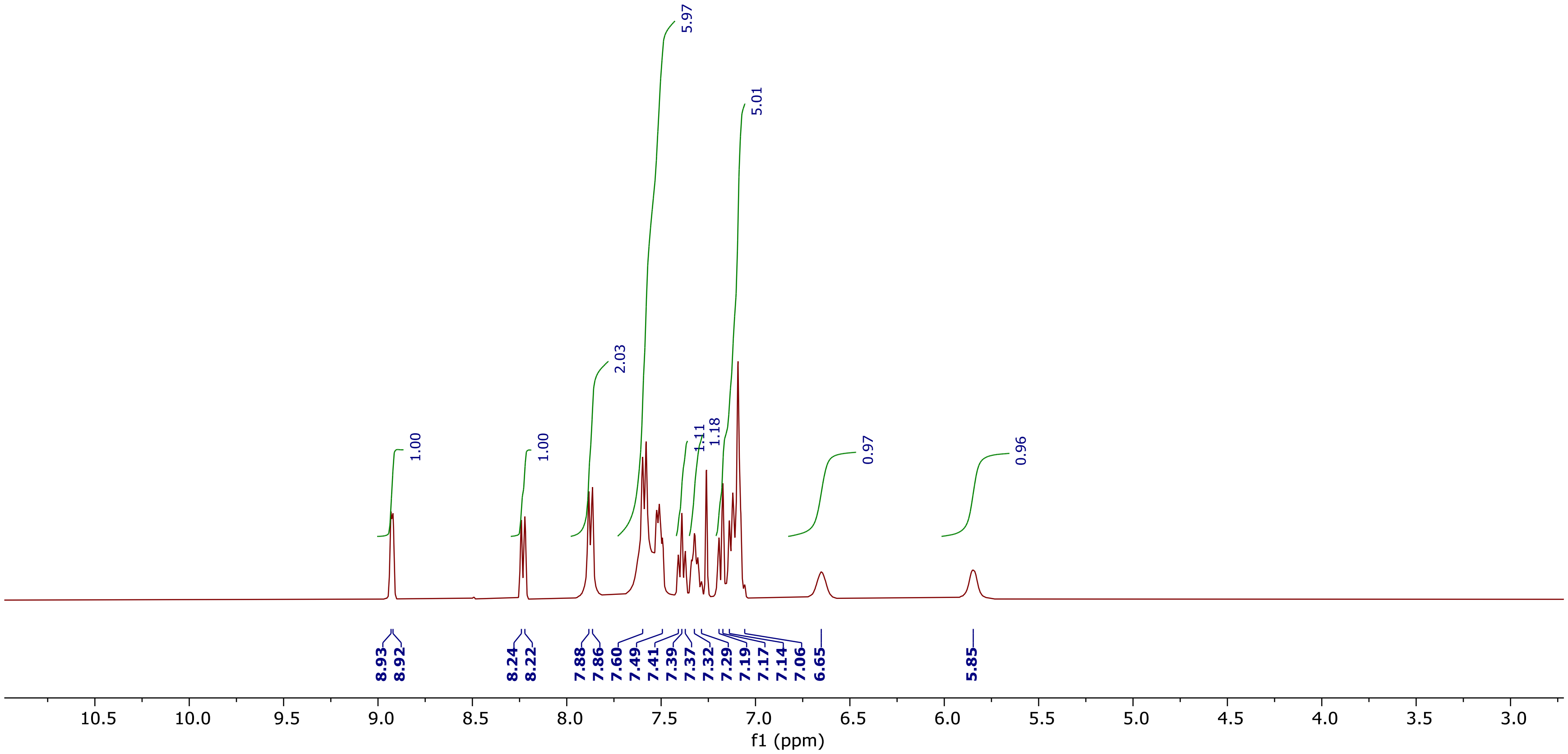

Figure S4. ^{1}H NMR spectrum of compound **4** (300 MHz, $CDCl_3$).

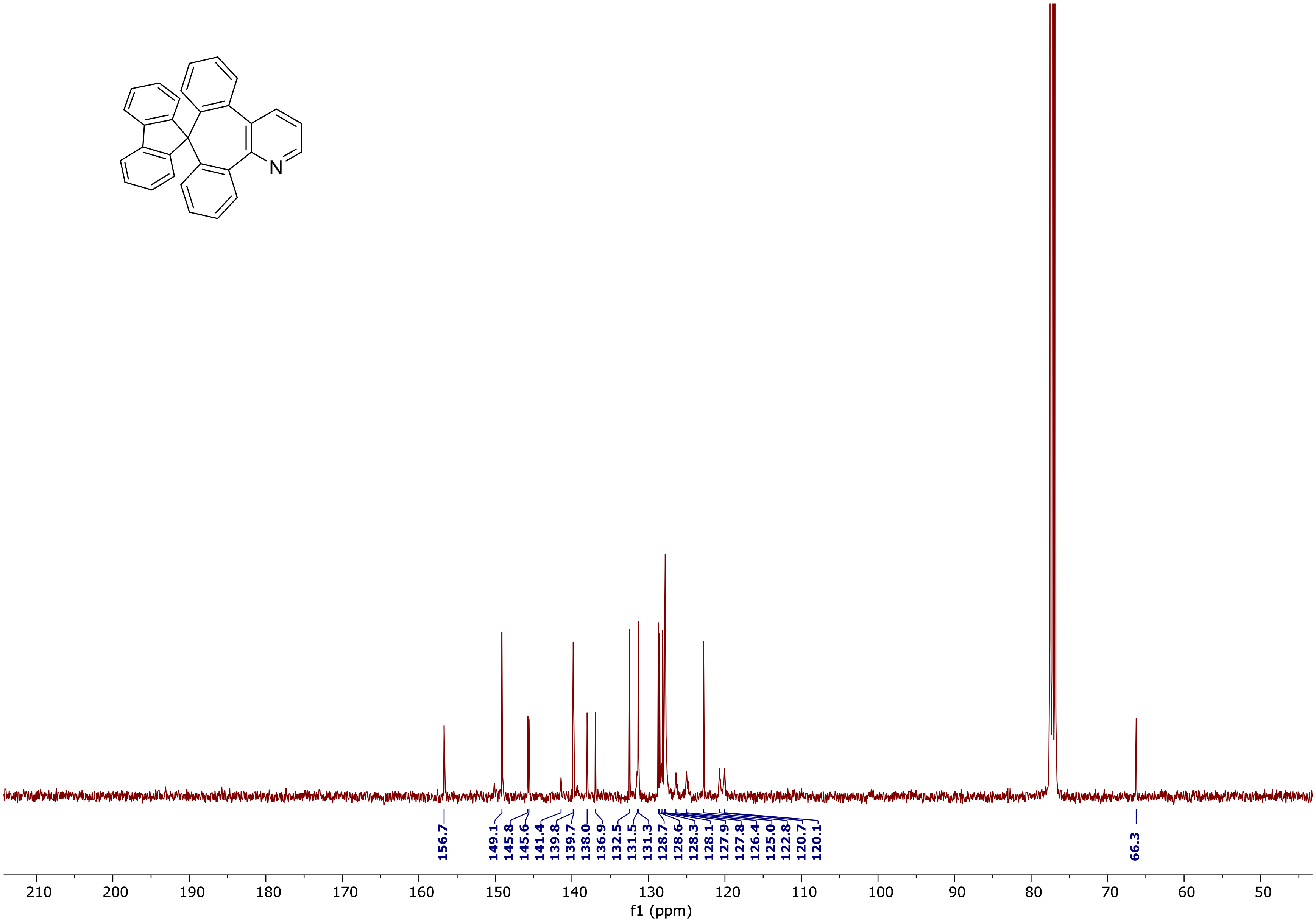

Figure S5. ^{13}C NMR spectrum of compound **4** (100.6 MHz, $CDCl_3$).

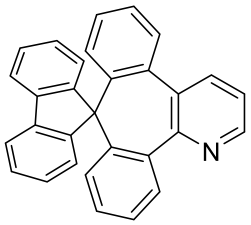

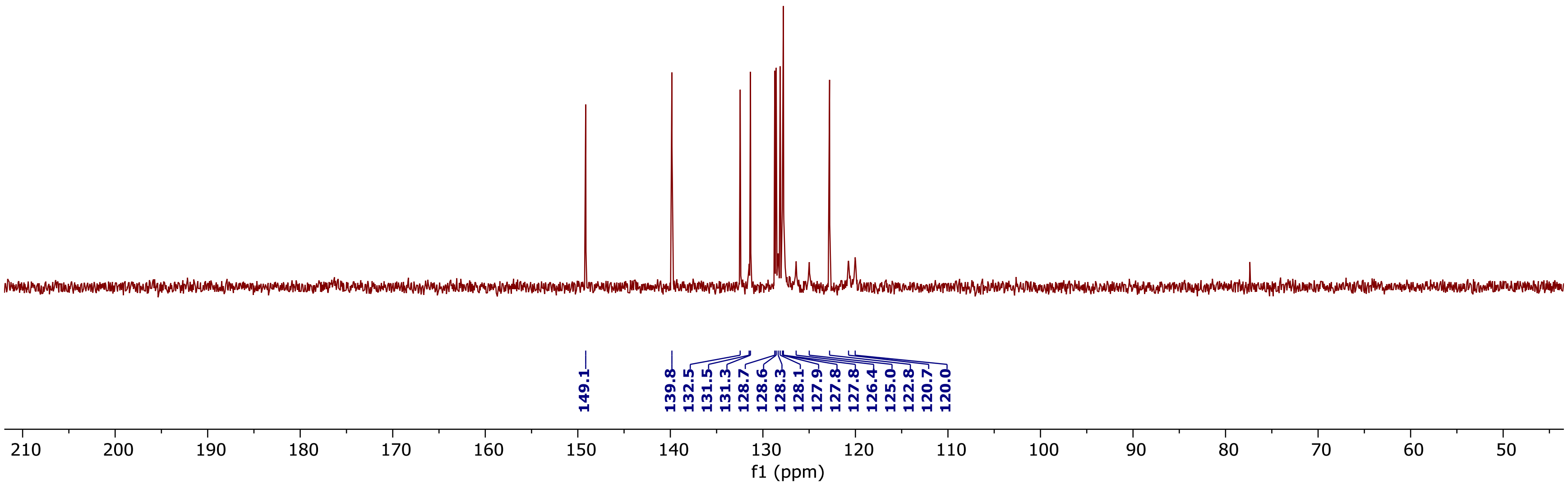

Figure S6. DEPT-135 ^{13}C NMR spectrum of compound **4** (100.6 MHz, $CDCl_3$).

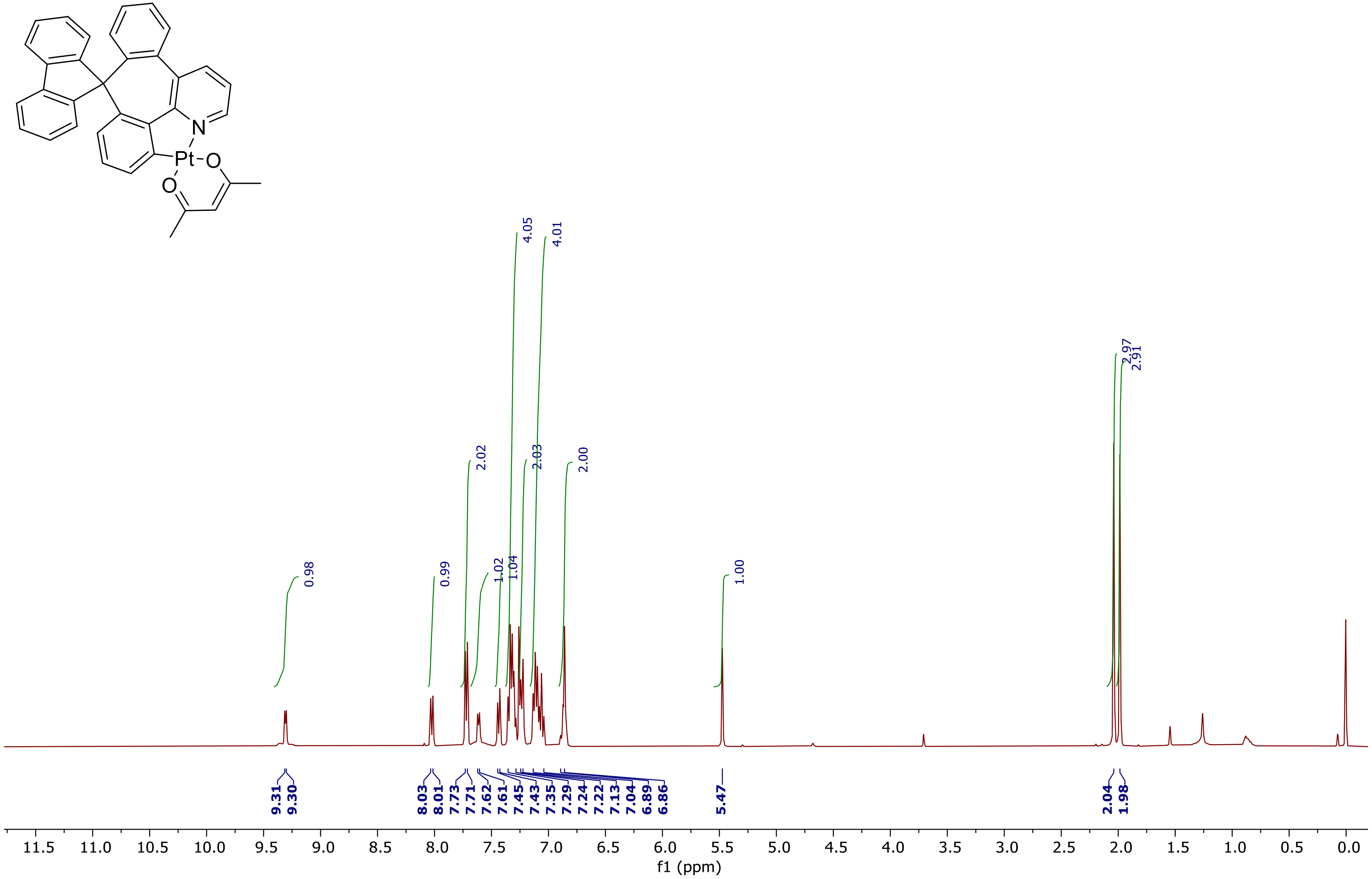

Figure S7. ^{1}H NMR spectrum of compound **7** (300 MHz, $CDCl_3$).

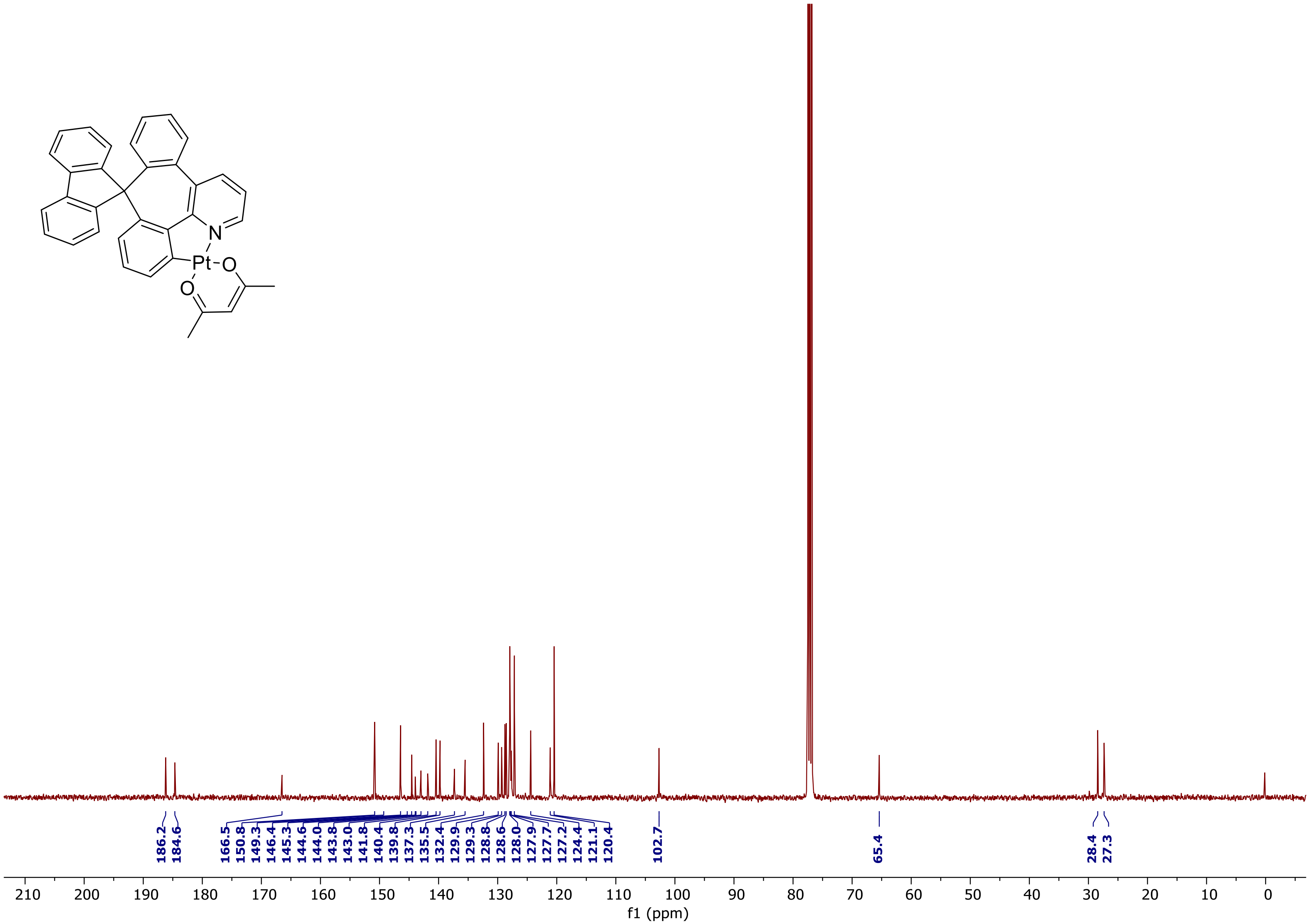

Figure S8. ^{13}C NMR spectrum of compound **7** (100.6 MHz, $CDCl_3$).

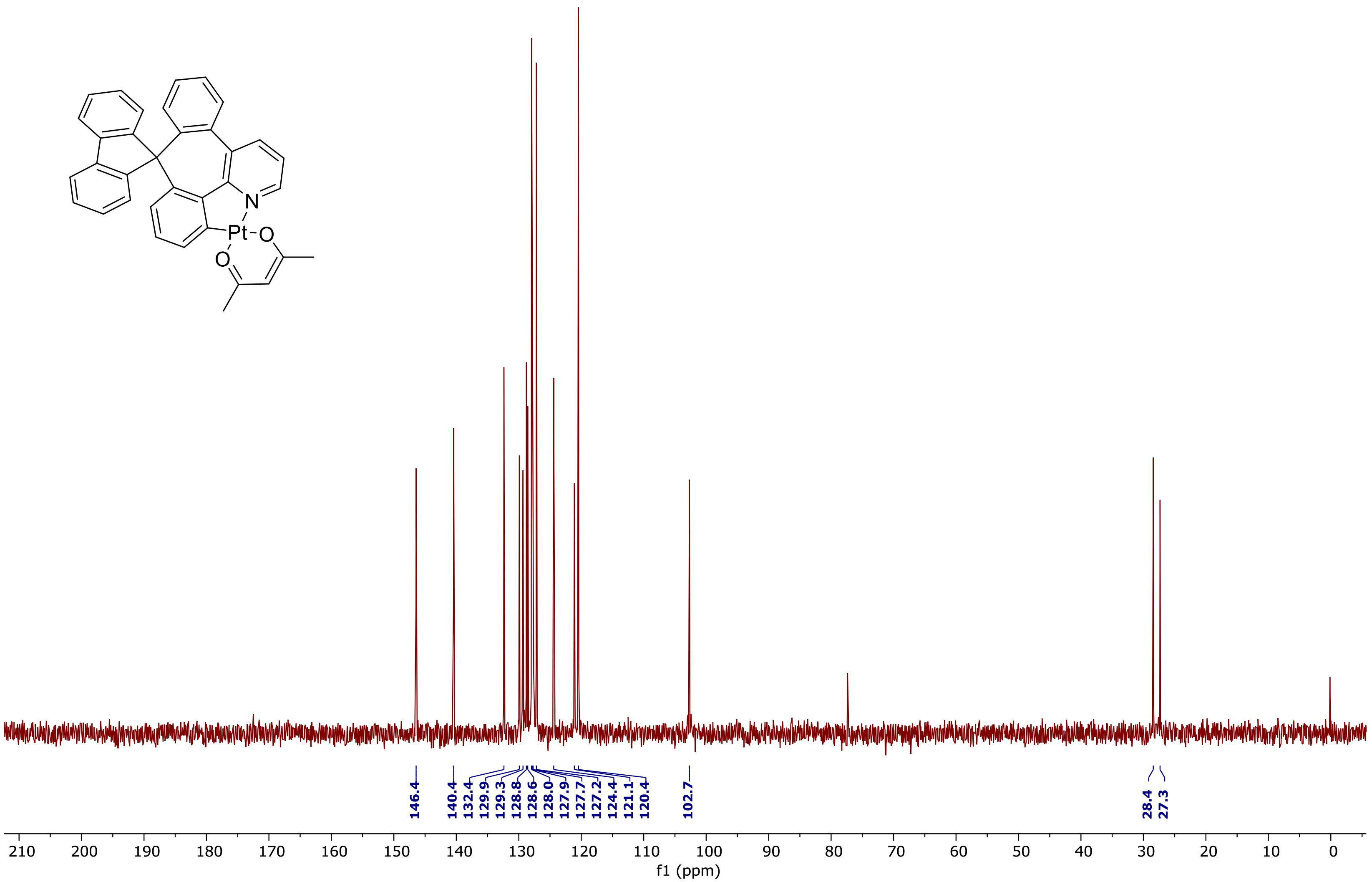

Figure S9. DEPT-135 ^{13}C NMR spectrum of compound **7** (100.6 MHz, $CDCl_3$).

4. Photophysics

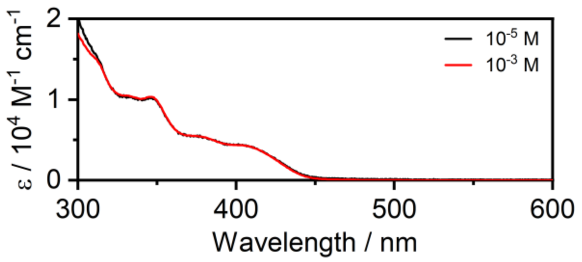

Figure S10. Comparison of absorption spectrum of **7** recorded at c = 10^{-5} M and at c = 10^{-3} M.

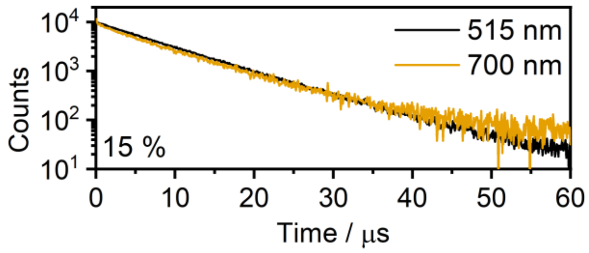

Figure S11. Photoluminescence decay traces for **7** 15% in PVK:PBD film at RT.

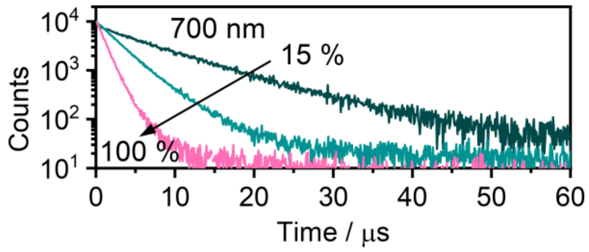

Figure S12. Photoluminescence decay traces for **7** films in PVK:PBD film at various concentrations at RT.

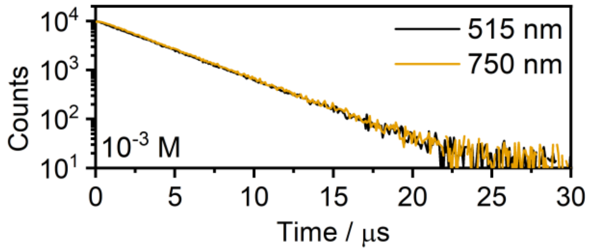

Figure S13. Photoluminescence decay traces in solution of **7** at c = 10^{-3} M.

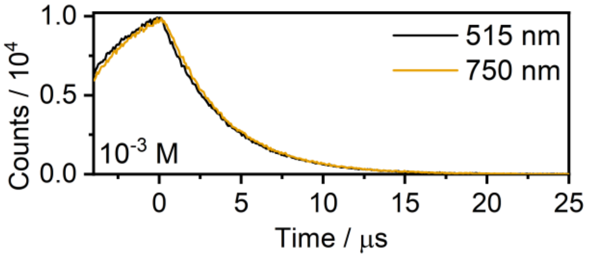

Figure S14. Photoluminescence decay traces in solution of **7** at c = 10^{-3} M in linear intensity scale.

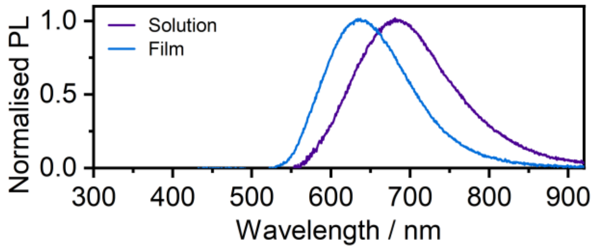

Figure S15. Comparison of decomposed PL spectra of **7** excimer/dimer species in film and solution.

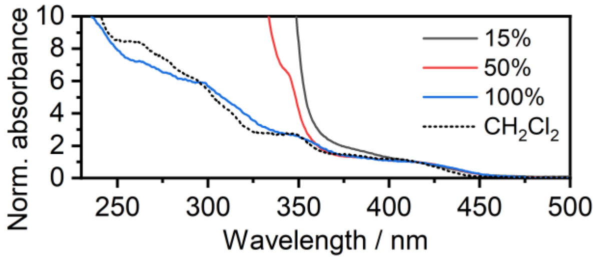

Figure S16. Absorption spectra in PVK:PBD films and neat film of **7** normalised to the absorption band at λ_{abs} = 415 nm. Note that the strong absorption at <350 nm for 15% and 50% film is due to the absorption of the host PVK:PBD matrix.

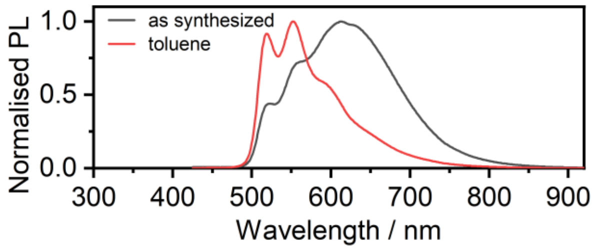

Figure S17. Normalised photoluminescence spectra of **7** in powder: (1) as obtained from the synthetic procedure (black line); (2) obtained from a slowly evaporated toluene solution (red line).

5. Electrochemical data

Table S1. Summary of electrochemical redox potentials recorded for **7** with cyclic voltammetry at a scan rate of 50 mV s^{-1}.

E_{onset}^{ox}, V[a]	E_{onset}^{red}, V[b]	IP, eV[c]	EA, eV[d]
0.67	-2.21	5.77	2.89

[a] Oxidation onset potential, V; [b] Reduction onset potential, V; [c] Ionization potential, IP = e[E_{onset}^{ox}] + 5.1, eV; [d] Electron affinity, IP = e[E_{onset}^{red}] + 5.1, eV.

6. Quantum chemical calculations

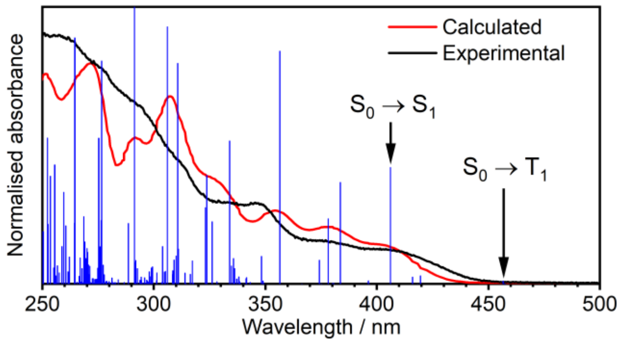

Figure S18. Calculated and experimental absorption spectra and simulated excitations in **7**.

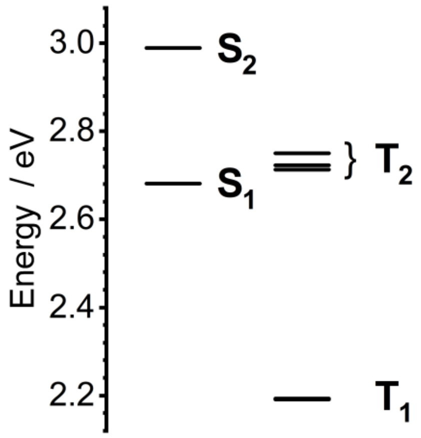

Figure S19. Excited states of **7** calculated at the T_1 geometry. Note that the splitting of the triplet levels is also shown and the split sublevels are cumulatively assigned to the principal excited state.

Table S2. Critical bond lengths at S_0 and T_1 geometry.

Bond length, Å	S_0	T_1
**Pt-N**	2.004	1.988
**Pt-C**	1.976	1.942
**Pt-O (I)**	2.124	2.120
**Pt-O (II)**	2.026	2.042

Table S3. Critical bond angles at S_0 and T_1 geometry.

Bond angle, °	S_0	T_1
**C-Pt-N**	80.4	81.7
**O-Pt-O**	90.1	89.9
**Pt-O-C (I)**	124.5	124.7
**Pt-O-C (II)**	125.9	125.7

Table S4. Critical dihedral angles at S_0 and T_1 geometry.

Dihedral angle, °	S_0	T_1
**O-O-C-N (Pt coordination plane)**	3.1	-0.7
**C-C(sp3 spiro)-C-C**	113.1	118.6

7. Electroluminescent devices

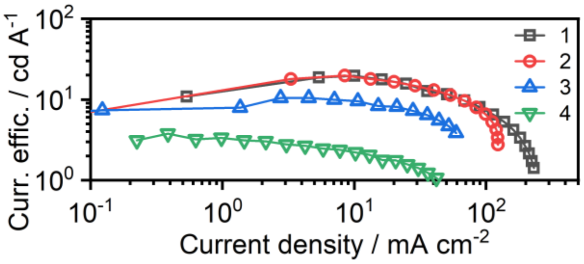

Figure S20. Characteristics of OLEDs 1-4: current efficiency *vs* current density.

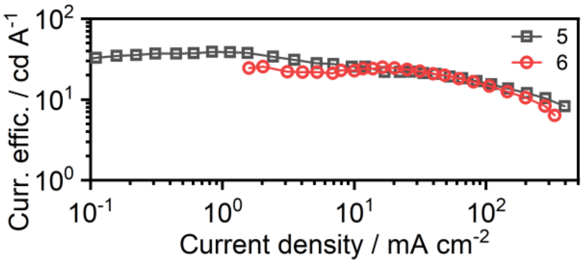

Figure S21. Characteristics of OLEDs 5 and 6: current efficiency *vs* current density.

Table S5. Structures of OLED devices.

**Device**	**Structure**
**Dev 1**	ITO | PEDOT AI 4083 (30 nm) | PVKH (10 nm) | PVK:PBD (60:40) co 5% **7** (30 nm) | TPBi (50 nm) | LiF (0.8 nm) | Al (100 nm)
**Dev 2**	ITO | PEDOT AI 4083 (30 nm) | PVKH (10 nm) | PVK:PBD (60:40) co 15% **7** (30 nm) | TPBi (50 nm) | LiF (0.8 nm) | Al (100 nm)
**Dev 3**	ITO | PEDOT AI 4083 (30 nm) | PVKH (10 nm) | PVK:PBD (60:40) co 50% **7** (30 nm) | TPBi (50 nm) | LiF (0.8 nm) | Al (100 nm)
**Dev 4**	ITO | PEDOT AI 4083 (30 nm) | PVKH (10 nm) | **7** (30 nm) | TPBi (50 nm) | LiF (0.8 nm) | Al (100 nm)
**Dev 5**	ITO | HAT-CN (10 nm) | TSBPA (40 nm) | mCP (2 nm) | mCP:T2T (60:40) co 10% **7** (20 nm) | T2T (2 nm) | TPBi (50 nm) | LiF (0.8 nm) | Al (100 nm)
**Dev 6**	ITO | HAT-CN (10 nm) | TSBPA (40 nm) | mCP (2 nm) | mCP:PO-T2T (80:20) co 10% **7** (20 nm) | PO-T2T (5 nm) | TPBi (40 nm) | LiF (0.8 nm) | Al (100 nm)

8. References

(1) Jou, J.-H.; Su, Y.-T.; Hsiao, M.-T.; Yu, H.-H.; He, Z.-K.; Fu, S.-C.; Chiang, C.-H.; Chen, C.-T.; Chou, C.; Shyue, J.-J. Solution-Process-Feasible Deep-Red Phosphorescent Emitter. *J. Phys. Chem. C* **2016**, *120*, 18794–18802.

(2) Roemelt, M.; Maganas, D.; DeBeer, S.; Neese, F. A Combined DFT and Restricted Open-Shell Configuration Interaction Method Including Spin-Orbit Coupling: Application to Transition Metal L-Edge X-Ray Absorption Spectroscopy. *J. Chem. Phys.* **2013**, *138*, 204101.

(3) de Souza, B.; Farias, G.; Neese, F.; Izsák, R. Predicting Phosphorescence Rates of Light Organic Molecules Using Time-Dependent Density Functional Theory and the Path Integral Approach to Dynamics. *J. Chem. Theory Comput.* **2019**, *15*, 1896–1904.

(4) Lenthe, E. van; Baerends, E. J.; Snijders, J. G. Relativistic Regular Two-component Hamiltonians. *J. Chem. Phys.* **1993**, *99*, 4597–4610.

(5) van Lenthe, E.; Baerends, E. J.; Snijders, J. G. Relativistic Total Energy Using Regular Approximations. *J. Chem. Phys.* **1994**, *101*, 9783–9792.

(6) Neese, F. The ORCA Program System. *WIREs Comput. Mol. Sci.* **2012**, *2*, 73–78.

(7) Neese, F. Software Update: The ORCA Program System—Version 5.0. *WIREs Comput. Mol. Sci.* **2022**, *12*.

(8) Becke, A. D. Density-functional Thermochemistry. III. The Role of Exact Exchange. *J. Chem. Phys.* **1993**, *98*, 5648–5652.

(9) Stephens, P. J.; Devlin, F. J.; Chabalowski, C. F.; Frisch, M. J. Ab Initio Calculation of Vibrational Absorption and Circular Dichroism Spectra Using Density Functional Force Fields. *J. Phys. Chem.* **1994**, *98*, 11623–11627.

(10) Weigend, F.; Ahlrichs, R. Balanced Basis Sets of Split Valence, Triple Zeta Valence and Quadruple Zeta Valence Quality for H to Rn: Design and Assessment of Accuracy. *Phys. Chem. Chem. Phys.* **2005**, *7*, 3297.

(11) Allouche, A.-R. Gabedit-A Graphical User Interface for Computational Chemistry Softwares. *J. Comput. Chem.* **2011**, *32*, 174–182.

(12) Neese, F.; Wennmohs, F.; Hansen, A.; Becker, U. Efficient, Approximate and Parallel Hartree–Fock and Hybrid DFT Calculations. A 'Chain-of-Spheres' Algorithm for the Hartree–Fock Exchange. *Chem. Phys.* **2009**, *356*, 98–109.

(13) Izsák, R.; Neese, F. An Overlap Fitted Chain of Spheres Exchange Method. *J. Chem. Phys.* **2011**, *135*, 144105.

(14) Weigend, F. Accurate Coulomb-Fitting Basis Sets for H to Rn. *Phys. Chem. Chem. Phys.* **2006**, *8*, 1057.

(15) Grimme, S.; Ehrlich, S.; Goerigk, L. Effect of the Damping Function in Dispersion Corrected Density Functional Theory. *J. Comput. Chem.* **2011**, *32*, 1456–1465.

(16) Grimme, S.; Antony, J.; Ehrlich, S.; Krieg, H. A Consistent and Accurate Ab Initio Parametrization of Density Functional Dispersion Correction (DFT-D) for the 94 Elements H-Pu. *J. Chem. Phys.* **2010**, *132*, 154104.

(17) Pantazis, D. A.; Chen, X. Y.; Landis, C. R.; Neese, F. All-Electron Scalar Relativistic Basis Sets for Third-Row Transition Metal Atoms. *J. Chem. Theory Comput.* **2008**, *4*, 908–919.

(18) Data, P.; Pander, P.; Lapkowski, M.; Swist, A.; Soloducho, J.; Reghu, R. R.; Grazulevicius, J. V. Unusual Properties of Electropolymerized 2,7- and 3,6- Carbazole Derivatives. *Electrochim. Acta* **2014**, *128*, 430–438.

(19) Pander, P.; Data, P.; Turczyn, R.; Lapkowski, M.; Swist, A.; Soloducho, J.; Monkman, A. P. Synthesis and Characterization of Chalcogenophene-Based Monomers with Pyridine Acceptor Unit. *Electrochim. Acta* **2016**, *210*, 773–782.

(20) Cardona, C. M.; Li, W.; Kaifer, A. E.; Stockdale, D.; Bazan, G. C. Electrochemical Considerations for Determining Absolute Frontier Orbital Energy Levels of Conjugated

Polymers for Solar Cell Applications. *Adv. Mater.* **2011**, *23*, 2367–2371.
(21) Bredas, J.-L. Mind the Gap! *Mater. Horiz.* **2014**, *1*, 17–19.
(22) de Sa Pereira, D.; Monkman, A. P.; Data, P. Production and Characterization of Vacuum Deposited Organic Light Emitting Diodes. *J. Vis. Exp.* **2018**, No. 141.
